# PTK6 inhibits autophagy to promote uveal melanoma tumorigenesis by binding to SOCS3 and regulating mTOR phosphorylation

**DOI:** 10.1038/s41419-023-05590-w

**Published:** 2023-01-23

**Authors:** Bo Liu, Xueting Yao, Chaoyang Zhang, Yufen Liu, Li Wei, Qinying Huang, Mengting Wang, Yanchen Zhang, Danning Hu, Wencan Wu

**Affiliations:** 1 State Key Laboratory of Ophthalmology, Optometry and Vision Science, Wenzhou Medical University, Wenzhou, China; 2 The Eye Hospital, School of Ophthalmology & Optometry, Wenzhou Medical University, Wenzhou, China; 3 Department of Laboratory Medicine, Longhua Hospital, Shanghai University of Traditional Chinese Medicine, Shanghai, China; 4 Department of General Surgery, The First Affiliated Hospital of Anhui Medical University, Hefei, China; 5 Tissue Culture Center, The New York Eye and Ear Infirmary, New York Medical College, Valhalla, New York, USA

**Keywords:** Oncogenes, Autophagy

## Abstract

Autophagy dysfunction is one of the common causes of tumor formation and plays an important role in uveal melanoma (UM). However, little is known about the regulatory mechanisms of autophagy in UM. Here, we show that PTK6 can promote the proliferation, migration, and invasion of UM cells by inhibiting autophagy. SOCS3 can inhibit the proliferation, migration, and invasion of UM cells. Overexpression of SOCS3 can partially rescue the PTK6-induced promotion of UM cell proliferation, migration, and invasion. Mechanistically, PTK6 can bind to SOCS3, and SOCS3 can downregulate the expression of PTK6. Furthermore, PTK6 can upregulate the phosphorylation of mTOR to inhibit autophagy. Taken together, our findings demonstrate the functions of PTK6 and SOCS3 in UM cells and targeting the SOCS3-PTK6 signaling axis might be a novel and promising therapeutic strategy for patients with UM.

## Introduction

Uveal melanoma (UM) is one of the most common malignant tumors of the eye and originates from melanocytes in the choroid, ciliary body, and iris. UM occurs frequently in the choroid, so many scholars have characterized choroidal melanoma as all types of UM [1]. According to the American Joint Committee of Cancer (AJCC), choroidal melanoma is classified into three cell types: (a) spindle cell type; (b) epithelioid cell type; (c) mixed cell type. Other rare variants of choroidal melanoma include (a) diffuse melanoma; (b) clear cell; (c) balloon cell melanoma [2, 3]. In recent decades, the mean annual incidence of UM has remained stable at 5 to 10 cases per million individuals [4]. Although significant progress has been made in radiotherapy and enucleation for UM patients, the overall survival rate of patients is still poor. The main causes of poor prognosis in patients with UM are metastasis and gene mutations. It has been reported that up to 50% of UM patients develop metastases, most commonly in the liver [5]. In addition, approximately 80% of somatic mutations affect GNAQ and GNA11 in UM patients [6], which leads to the overactivation of G protein-coupled receptors (GPCRs), followed by activation of the downstream mitogen-activated protein kinase (MAPK) pathway [7, 8]. Therefore, screening new molecular markers for UM patients is still a high priority.

Autophagy is a normal physiological process of clearing cellular damaged proteins and organelles through the formation of autophagosomes. It facilitates the degradation of abnormal components to maintain a normal cellular steady-state environment, which is conducive to cell survival, differentiation, and development [9, 10]. Dysregulation of autophagy is associated with the development of various diseases, including cancers [11]. Autophagy is a double-edged sword in cancers. Whether autophagy promotes or inhibits tumors might depend on the stage. Autophagy can eliminate abnormal components to maintain cellular structure and metabolic stability in the early stages of tumor formation, which suppresses the development of tumors [12]. In contrast, autophagy overactivation promotes tumor growth by allowing tumor cells to absorb energy from degraded cellular damaged proteins and organelles in the advanced stages of tumor formation [13]. The phenomenon of autophagy is also regulated by various signaling pathways. For example, the PI3K/AKT/mTOR, p53, MAPK, NFkB, and other signaling pathways can affect the survival of tumor cells by interacting with autophagy [9, 14, 15]. Therefore, it is very complicated to treat tumors by regulating autophagy. The role of autophagy in UM has been reported. For example, the long noncoding RNA ZNNT1 induces autophagy to inhibit tumorigenesis of UM by regulating ATG12 expression [16]; mutant GNAQ can induce AMPK-dependent autophagic cell death in UM [17]; and autophagy-related long noncoding RNA can evaluate the prognosis of UM patients [18]. Meanwhile, it has been reported that the expressions of three autophagy-related proteins (Beclin-1, p62, and ATG7) are closely related to clinicopathological parameters and blood vascular microvessel density and UM patients with high expression of Beclin-1 have more positive prognostic value [19]. However, studies of the mechanism of autophagy regulation in UM cells are still limited.

To analyze the mechanism of autophagy in UM, protein tyrosine kinase 6 (PTK6) was screened from the Cancer Genome Atlas (TCGA) database through the prognosis of melanoma patients and the difference of protein expression in UM. PTK6, also known as breast tumor kinase (BRK), is a nonreceptor intracellular tyrosine kinase cloned from metastatic breast cancers that can promote the differentiation of normal epithelial cells [20, 21]. PTK6 can promote tumorigenesis of breast cancer, lung cancer, colorectal cancer, and other tumors [22–24]. However, the effect of PTK6 on UM has not been reported. Mechanistically, PTK6 can influence the activity of JAK2/STAT3, PTEN, EGFR, and other pathways [22, 24, 25]. Little is known about the function of PTK6 in the autophagy pathway. Therefore, we hypothesized that PTK6 promotes UM tumorigenesis by inhibiting autophagy.

In this study, we found that PTK6 promoted the proliferation, migration, and invasion of UM cells in vitro and in vivo. The knockdown of PTK6 promoted autophagy upon inhibiting the phosphorylation of mTOR protein. The overexpression of PTK6-inhibited autophagy upon promoting the phosphorylation of mTOR protein. At the same time, we further demonstrated that PTK6 promotes tumorigenesis of UM by inhibiting autophagy based on the experimental results of the autophagy agonist rapamycin and autophagy inhibitor 3-methyladenine (3-MA). Our study revealed a new molecular mechanism of PTK6, which might potentially have therapeutic value in autophagy dysfunction-mediated UM.

## Materials and methods

### Analysis of online database

The clinical and RNA-Seq data of patients were downloaded from the TCGA database (https://portal.gdc.cancer.gov/). The RNA-Seq data of normal tissues were downloaded from the Genotype-Tissue Expression (GTEx) database (https://xena.ucsc.edu/). The 232 human autophagy-related genes (ARGs) were obtained from the Human Autophagy Database (http://autophagy.lu/clustering/index.html). The methods of bioinformatic analysis were described previously [26, 27].

### Cell lines and culture

The UM cell lines C918 and MUM-2B were purchased from FuHeng Cell Center, Shanghai, China. The cells were cultured in RPMI 1640 medium (Thermo Fisher, C11875500BT) supplemented with 10% fetal bovine serum (Lonicera, S711-001S). The UM cell line SP6.5 was obtained from the group of Dan-Ning Hu (Icahn School of Medicine at Mount Sinai, New York, NY, USA). The SP6.5 cell line was cultured in DMEM supplemented with 10% FBS. The melanoma cell line A375 cultured in DMEM supplemented with 10% FBS was also purchased from FuHeng Cell Center, Shanghai, China. All cell lines were tested by STR analysis. The primary uveal melanocyte cell line U-94 was obtained from a donor at Wenzhou Medical University and cultured as previously described [28, 29]. The ARPE-19 cell line was purchased from Procell Life Science & Technology Corp., Ltd., Wuhan, China, and cultured in F12/DMEM (Gibco) supplemented with 10% FBS. The use of human tissue in this study was approved by the Wenzhou Medical University ethics committee.

### Immunofluorescence

For immunostaining, cells were fixed in 4% paraformaldehyde (PFA) for 30 min and blocked using 3% BSA at room temperature for 1 h. Primary antibodies were anti-PTK6 (1:200; CST, 55174 S), anti-SOCS3 (1:200; Proteintech, 66797-1), anti-LC3 (1:200; Abcam, ab192890), and anti-DAPI (1:1000; Beyotime, C1002). Secondary antibodies (anti-mouse or anti-rabbit Alexa 594-conjugated or Alexa 488-conjugated donkey) were applied at room temperature for 1 h for detection.

### Western blotting and quantitative real-time PCR

The steps of Western blotting and quantitative real-time PCR were as previously described [30, 31]. For Western blotting, the primary antibodies were anti-GAPDH (1:1000; Bioss, bsm-0978M), anti-PTK6 (1:1000; Abcam, ab233392), anti-SOCS3 (1:1000; Abcam, ab16030), anti-BIRC5 (1:1000; OriGene, TA501245S), anti- EGFR (1:1000; OriGene, TA506224S), anti- DAPK2 (1:1000; OriGene, TA501099S), anti- ATG9B (1:1000; HuaBio, ER61710), anti- IFNG (1:1000; Beyotime, AF7173), anti-LC3 (1:1000; Abcam, ab192890), anti-P62 (1:1000; Abcam, ab109012), anti-P62 (1:1000; CST, 23214), anti-mTOR (1:1000; Abcam, ab134903), and anti-p-mTOR (1:1000; Abcam, ab109268). The primers for Q-PCR were as follows: *GAPDH*: F: AATGGGCAGCCGTTAGGAAA, R: GCCCAATACGACCAAATCAGAG; *PTK6*: F: ATGTGCCCCACAACTACCTG, R: TAGTTCACAAGCTCGGGCAG; *SOCS3*: F: CGGACCTGGAATGTGTTGGA, R: TCAGCATTCCCGAAGTGTCC; *BIRC5*: F: GATGACGACCCCATGCAAAG, R: GTGGCACCAGGGAATAAACC; *IFNG*: F: GCTCTGCATCGTTTTGGGTTC, R: TTTTCTGTCACTCTCCTCTTTCC; *EGFR*: F: CCTGAGCTCTCTGAGTGCAAC, R: GCTTGGACACTGGAGACTGG; *DAPK2*: F: GGTGCTCATCCTTGAGCTAGTG, R: TTCGTGAGCCAGACCAAAGT; and *ATG9B*: F: CGAGCTAAATGGAAGGGCGA, R: ACAAGTCCCCATCCCCTACA.

### Cell proliferation assays and cell treatment

SP6.5, A375, C918, and MUM-2B cells (2000 cells/well) were seeded in a 96-well plate. Cell Counting Kit-8 (CCK-8) reagent (1:11; Beyotime, C0038) was added to each well for 2 h. The optical density was measured at 450 nm. UM cells were treated with 30 μmol/L rapamycin (Apexbio, A8167) and 30 μmol/L 3-methyladenine (Apexbio, A8353) and cultured in RPMI 1640 medium supplemented without 10% FBS. Resveratrol was purchased from the company of Beyotime (Beyotime, SC0276).

### Transwell migration and invasion assay

The migration and invasion ability of UM cells was evaluated using a 24-well Transwell system (Corning, 3422, 354480). SP6.5, A375, C918, and MUM-2B cells (1 × 10^4^) were resuspended in serum-free RPMI 1640 medium and seeded into the upper chambers. RPMI 1640 medium with 20% FBS was added to the lower chambers. After 24 h, the Transwell chamber was fixed in 4% PFA for 10 min and stained with crystal violet (Beyotime, C0121) for 10 min.

### Xenografts assays

Four- to six-week-old BALB/c nude mice were purchased from Charles River Laboratories, China, and housed in the specific-pathogen-free (SPF) laboratory animal room at Wenzhou Medical University. Uveal melanoma cells (2 × 10^6^) cultured in 100 μL PBS were injected into the subcutaneous flank of the mice. The tumor size was measured every week with a caliper, and the tumor volume was calculated using the methods in the literature [32]. After 4 weeks, the excised tumors were weighed and photographed.

### Plasmid and small interfering RNA (siRNA)

The human *PTK6* coding sequence (CDS) (NM_005975) was cloned into the lentivirus Ubi-3xFlag vector. The human *SOCS3* CDS (NM_003955) was cloned into the lentivirus Enhancer-3xFlag vector. The vectors were purchased from GeneChem (Shanghai, China). The siRNA sequences of *PTK6* and *SOCS3* were synthesized by GenePharma (Shanghai, China) as follows: *Si-NC*: 5′-UUCUCCGAACGUGUCACGUTT-3′; *Si-PTK6-1*: 5′-GAGCUUGUGAACUACCACATT-3′; *Si-PTK6-2*: 5′-GGCCAUUACUCCACCAAAUTT-3′; *Si-SOCS3-1*: 5′-UCUUCACGCUCAGCGUCAATT-3′; and *Si-SOCS3-2*: 5′-GCCACUCUUCAGCAUCUCUTT-3′.

### Statistical analysis

Bioinformatic analysis was performed using R software with the chi-square test, univariate and multivariate analyses, or Kaplan–Meier analysis. The experimental data were presented as the mean ± standard deviation (SD). Statistical analyses were carried out using GraphPad Prism 8 with Student’s *t*-test when comparing two groups, and one-way or two-way ANOVA with Bonferroni post hoc test were performed for comparing more than two groups. *p* < 0.05 was considered to indicate statistical significance.

## Results

### Prognostic value of autophagy-related genes in melanoma

**Fig. 1:**
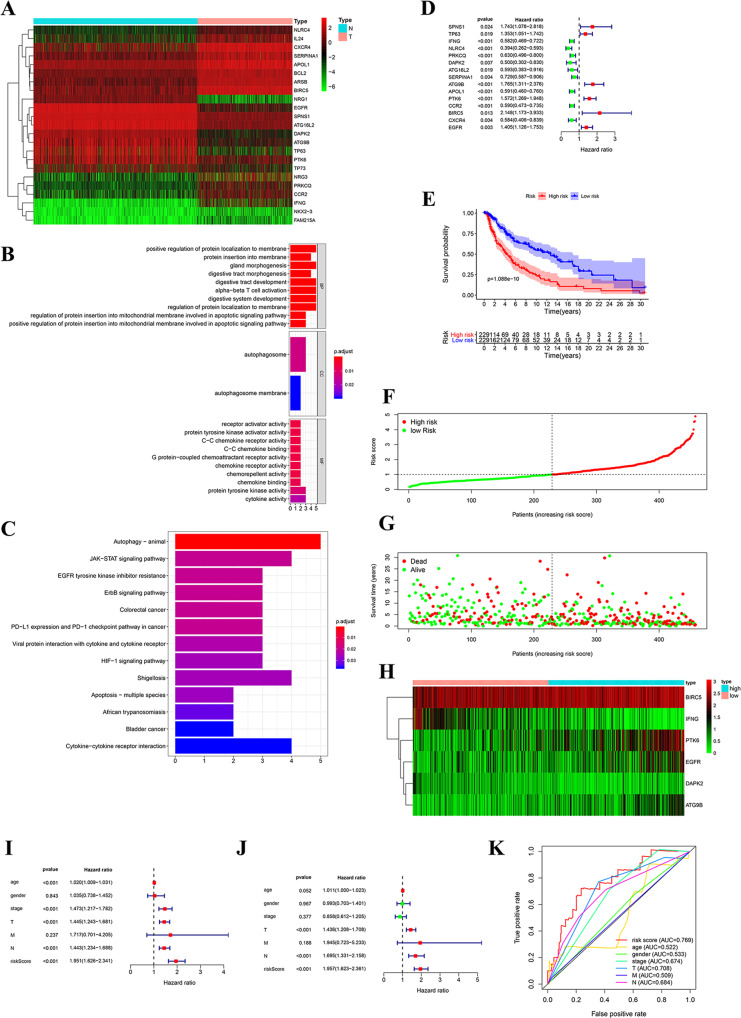
Prognostic value of ARGs in melanoma. **A** The 23 differentially expressed ARGs of melanoma. N represents normal and T represents tumor. **B**, **C** GO and KEGG enrichment analysis of differentially expressed genes. **D** Forest plot of the prognostic signature for predicting patient OS by univariate Cox regression analysis. **E** OS curves for melanoma patients in the high-risk group and the low-risk group. **F** The risk score of each patient. **G** The survival status of each patient. **H** The expression heatmap of the six ARGs. **I**, **J** The clinical parameters in the univariate and multivariate Cox regression analyses. **K** The ROC curve of the risk score and clinical parameters in predicting the 5-year survival rate of melanoma patients.

We analyzed the expression of 232 ARGs in 813 normal tissue and 471 melanoma samples, and 23 differentially expressed genes were screened out (|log_2_FC|> 1, adjusted *p* < 0.001) (Fig. 1A). Gene ontology (GO) and Kyoto Encyclopedia of Genes and Genomes (KEGG) enrichment analysis showed that 23 ARGs were mainly enriched in the autophagy pathway (Fig. 1B, C). This indicates that these genes might affect the autophagy pathway in melanoma. Fifteen ARGs were screened out and used to develop a prognostic signature for the overall survival (OS) of patients by univariate Cox regression analysis (*p* < 0.05). The forest map of 15 ARGs was shown (Fig. 1D). Then, we performed cox regression dimensionality reduction analysis to obtain six ARGs (IFNG, DAPK2, ATG9B, PTK6, BIRC5, and EGFR). We obtained the risk scores of patients with melanoma: risk score = (−0.379123977 × expression of IFNG) + (−0.662084468 × expression of DAPK2) + (0.342818269 × expression of ATG9B) + (0.406559238 × expression of PTK6) + (0.807218069 × expression of BIRC5) + (0.364523721 × expression of EGFR). All patients with melanoma were divided into high-risk and low-risk groups based on the median value of the risk score. As shown in Fig. 1E, the high-risk score melanoma patients had shorter survival (*p* < 0.001) than the low-risk group. Figure 1F–H shows the risk score of each patient (Fig. 1F), the survival status of each patient (Fig. 1G), and the expression heatmap for the six ARGs (Fig. 1H). Based on the univariate Cox regression analysis, patient age, tumor stage, T stage, N stage, and risk score (all *p* < 0.001) were associated with survival (Fig. 1I). Patient T, N, and risk score (all *p* < 0.001) were independent risk factors for survival according to multivariate Cox regression analysis (Fig. 1J). The receiver operating characteristic (ROC) curve indicated that the risk score (area under the curve; AUC = 0.769) was more accurate than the clinical parameters (age, sex, stage, T stage, N stage, and M) in predicting the 5-year survival rate of melanoma patients (Fig. 1K).

### PTK6 expression correlates with the survival of melanoma patients

According to the results of the above research, we further assessed the role of 6 ARGs in UM. We downloaded the clinical and RNA-Seq data of UM patients from the TCGA database. A correlation circle graph was generated based on the expression of six ARGs in UM (Fig. S1A). We also examined the expression of six ARGs in UM cells (C918, MUM-2B) and primary uveal melanocyte cells (U-94) (Fig. S1B, E). Melanoma patients with high expression of IFNG and DAPK2 have a better prognosis. However, IFNG and DAPK2 expressions were increased in UM cell lines compared to uveal melanocytes cell (Fig. S1B, E). Melanoma patients with high expression of BIRC5, EGFR, and ATG9B had a worse prognosis. Moreover, BIRC5, EGFR, and ATG9B expressions were increased in UM cell lines compared to uveal melanocytes cell (Fig. S1B, E). However, PTK6 was highly correlated with the 6 ARGs, and the expression of *PTK6* was also significantly increased in UM cells compared with U-94 cells (Fig. S1A, B, E). Furthermore, according to the differential expression of *PTK6* in melanoma patients, we divided the patients into a *PTK6* high-expression group and a *PTK6* low-expression group. Patients with high PTK6 expression had shorter survival than patients with low PTK6 expression (Fig. S1C). Finally, we found that the *PTK6* high-expression and low-expression data set were mainly enriched in signaling pathways closely related to tumors based on gene set enrichment analysis (GSEA) (Fig. S1D), suggesting that *PTK6* might promote the formation of UM.

### PTK6 promotes the proliferation, migration, and invasion of UM cells in vitro and in vivo

**Fig. 2:**
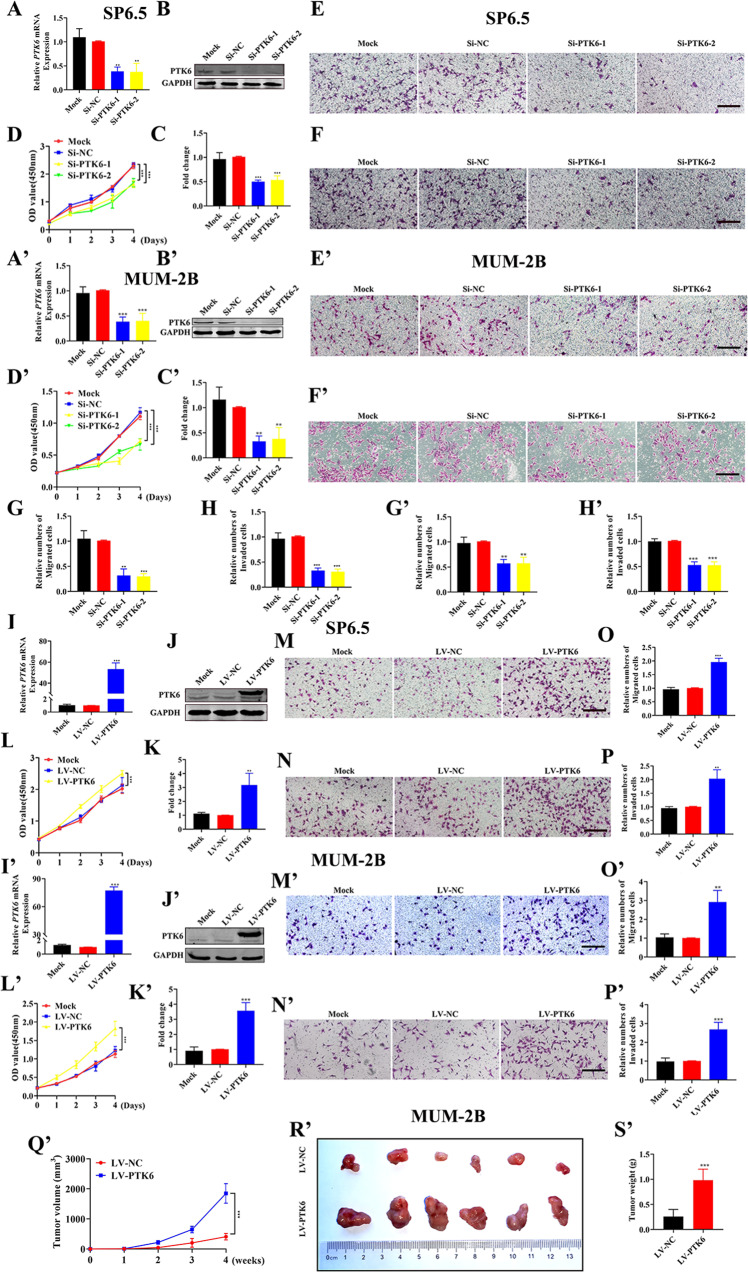
PTK6 promotes the proliferation, migration, and invasion of UM cells in vitro and in vivo. **A**, **A’** The expression of PTK6 mRNA in UM cells with PTK6 knockdown. **B**, **C** and **B’**, **C’** Western blotting analysis of PTK6 in UM cells with PTK6 knockdown. The above datas were analyzed using one-way ANOVA with Bonferroni. **D**–**H** and **D’**–**H’** Analysis of the proliferation, migration, and invasion of UM cells with PTK6 knockdown. **D** and **D**’ were analyzed using two-way ANOVA with Bonferroni. **E**–**H** and **E**’–**H**’ were analyzed using one-way ANOVA with Bonferroni. **I**, **I’** The expression of PTK6 mRNA in UM cells overexpressing PTK6. **J**, **K** and **J’**, **K’** Western blotting analysis of PTK6 in UM cells overexpressing PTK6. The above data were analyzed using one-way ANOVA with Bonferroni. **L**–**P** and **L’**–**P’** Analysis of the proliferation, migration, and invasion of UM cells overexpressing PTK6. **L** and **L**’ were analyzed using two-way ANOVA with Bonferroni. **M**–**P** and **M’**–**P’** were analyzed using one-way ANOVA with Bonferroni. **Q’** The volume of tumors formed in the LV-NC group and LV-PTK6 group. The data were analyzed using two-way ANOVA with Bonferroni. **R’** Photographic images of tumors from the LV-NC group and LV-PTK6 group. **S’** The weight of tumors formed in the LV-NC group and LV-PTK6 group. The data were analyzed using Student’s *t*-test. (A-P: SP6.5 cell; **A**’–**S**’: MUM-2B cell; scale bar: 100 µm; Data were presented as the mean ± SD; *n* = 3–6; *p < 0.05, **p < 0.01, ***p < 0.001).

As shown in Fig. S2, the expression of PTK6 in SP6.5, C918, and MUM-2B UM cell lines were statistically more valuable than OCM1, so we chose the three cell lines for the subsequent research. To investigate the role of *PTK6* in UM, we knocked down the expression of *PTK6*. As shown in Fig. 2A–C, A’–C’ and S4A–C, *PTK6* was successfully knocked down in both SP6.5, MUM-2B, and C918 cell lines, and the expression of PTK6 was decreased by more than 50% in the *Si-PTK6* groups compared with the *Si-NC* groups. Then, we examined the proliferation, migration, and invasion of UM cells. As shown in Fig. 2D–H, D’–H’ and S4D–H, in SP6.5, MUM-2B and C918 cells, the proliferation, migration, and invasion abilities of the *Si-PTK6* group cells were decreased compared with those of the *Si-NC* group cells. The data suggested that knocking down *PTK6* could inhibit tumorigenesis of UM cells. Similarly, we overexpressed *PTK6* in SP6.5, MUM-2B, and C918 cells using lentivirus technology. As shown in Fig. 2I–K, I’–K’ and S4I–K, *PTK6* was stably overexpressed in UM cells. In contrast, the proliferation, migration, and invasion abilities of UM cells overexpressing PTK6 (LV-PTK6) were stronger than those of the control cells (LV-NC) (Fig. 2L–P, L’–P’ and S4L–P). Finally, we injected LV-PTK6 and LV-NC cells subcutaneously into nude mice and examined the expression of PTK6 protein in the two groups of tumors (S14A, D). As shown in Fig. 2Q’–S’ and S4Q–S, the tumors formed were significantly larger (in terms of volume and weight) in the LV-PTK6 group than in the LV-NC group. The above data demonstrated that PTK6 promotes UM tumorigenesis in vitro and in vivo. Meanwhile, we also knocked down and overexpressed PTK6 in the melanoma cell line (A375), and tested the ability of A375 proliferation, migration, and invasion (Fig. S3A–N). The data showed that PTK6 also promoted tumor growth in melanoma.

### PTK6 promotes the proliferation, migration, and invasion of UM cells by inhibiting autophagy

**Fig. 3:**
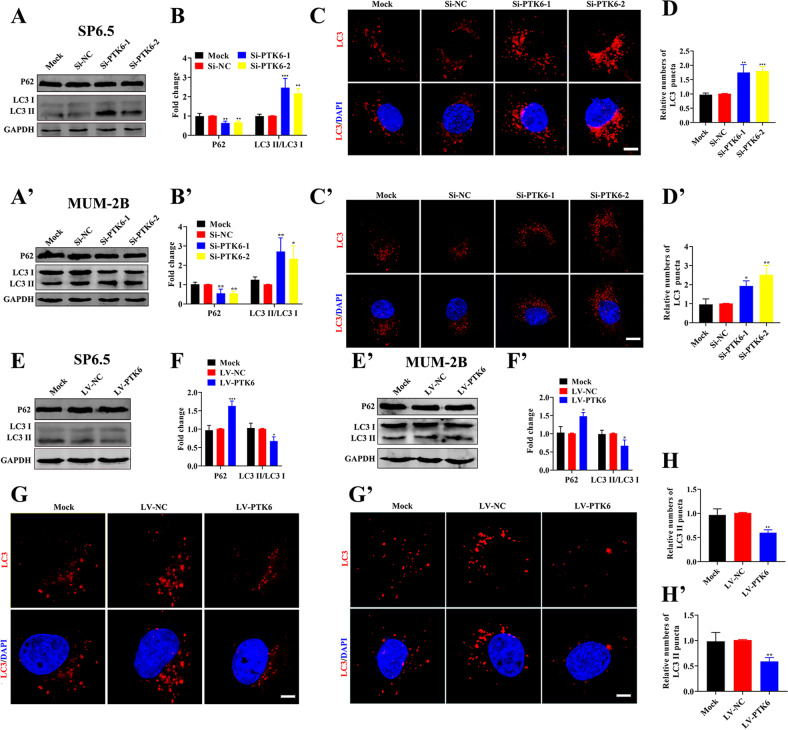
PTK6 inhibits autophagy in UM cells. **A**, **B** and **A’**, **B’** Western blotting analysis of LC3 and P62 in UM cells with PTK6 knockdown. **C**, **D** and **C’**, **D’** Immunofluorescence analysis of autophagosomes in UM cells with PTK6 knockdown. The above data were analyzed using one-way ANOVA with Bonferroni. **E**, **F** and **E’**, **F’** Western blotting analysis of LC3 and P62 in UM cells overexpressing PTK6. **G**, **H** and **G’**, **H’** Immunofluorescence analysis of autophagosomes in UM cells overexpressing PTK6. The above data were analyzed using one-way ANOVA with Bonferroni. (A-H: SP6.5 cell; **A**’–**H**’: MUM-2B cell; scale bar: 20 µm; Data were presented as the mean ± SD; *n* = 3; *p < 0.05, **p < 0.01, ***p < 0.001).

Then, we analyzed how PTK6 affects the proliferation, migration, and invasion of UM cells. We detected the expression of autophagosomes in UM cells with PTK6 knockdown or overexpression. As shown in Fig. 3A, B, A’, B’ and S5A, B, in SP6.5, MUM-2B, and C918 cells, the ratio of LC3 II/LC3 I in the *Si-PTK6* groups was higher than that in the *Si-NC* groups, and the expression of P62 was decreased. In addition, the number of autophagosomes in the *Si-PTK6* groups was also higher than that in the *Si-NC* groups according to immunofluorescence analysis (Fig. 3C, 3D, C’, D’ and S5C, D). This indicated that knocking down PTK6 in UM cells promoted the autophagy pathway. Similarly, as shown in Fig. 3E, F, E’, F’ and S5E, F, the ratio of LC3 II/LC3 I in the LV-PTK6 group was lower than that in the LV-NC group, the expression of P62 was increased, and the number of autophagosomes was also reduced. This indicated that the overexpression of PTK6 in UM cells inhibits the autophagy pathway.

**Fig. 4:**
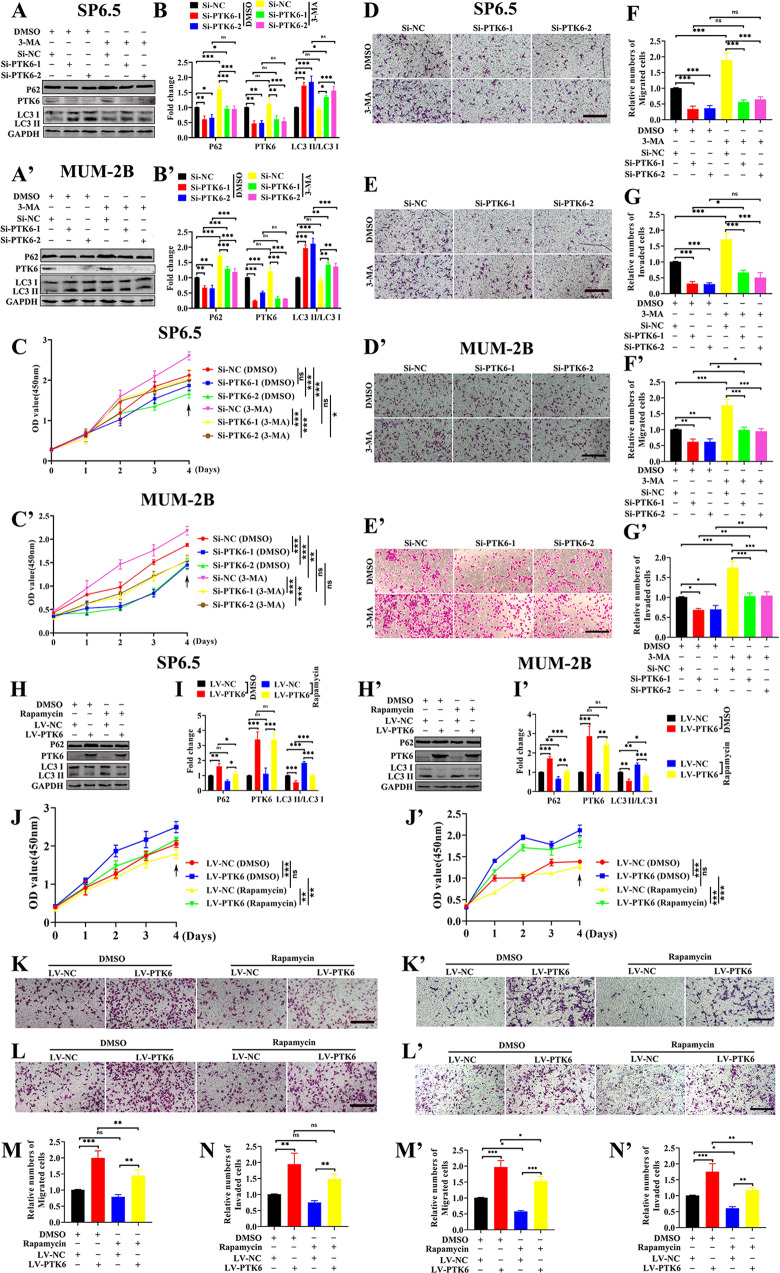
PTK6 promotes the proliferation, migration, and invasion of UM cells by inhibiting autophagy. **A**, **B** and **A’**, **B’** Western blotting analysis of LC3, PTK6, and P62 in the *Si-PTK6* groups and *Si-NC* group after treatment with DMSO or 3-MA. The data were analyzed using two-way ANOVA with Bonferroni. **C**–**G** and **C’**–**G’** Proliferation, migration, and invasion analyses of the *Si-PTK6* groups and *Si-NC* group after treatment with DMSO or 3-MA. The data were analyzed using two-way ANOVA with Bonferroni. **H**, **I** and **H’**, **I’** Western blotting analysis of LC3, PTK6, and P62 in the LV-NC group and LV-PTK6 group after treatment with DMSO or rapamycin. The data were analyzed using two-way ANOVA with Bonferroni. **J**–**N and J’**–**N’** Proliferation, migration, and invasion analyses of the LV-NC group and LV-PTK6 group after treatment with DMSO or rapamycin. The data were analyzed using two-way ANOVA with Bonferroni. **A**–**N**: SP6.5 cell; **A**’–**N**’: MUM-2B cell; scale bar: 100 µm; data were presented as the mean ± SD; *n* = 3; ns no significant difference, *p < 0.05, **p < 0.01, ***p < 0.001).

Next, we aimed to explore whether PTK6 affects the proliferation, migration, and invasion of UM cells through the autophagy pathway. According to the results of previous research [16], we explored the suitable concentrations of the autophagy agonist rapamycin and the autophagy inhibitor 3-MA in SP6.5, MUM-2B, and C918 cells. As shown in Fig. S6A–F, the suitable concentration of both rapamycin and 3-MA for this research was 30 μmol/L. We detected the changes in autophagosomes and the proliferation, migration, and invasion of UM cells by adding 3-MA or DMSO to UM cells with PTK6 knockdown. As shown in Fig. 4A, B, A’, B’ and S7A, B, in SP6.5, MUM-2B, and C918 cells, autophagy was increased in the *Si-PTK6* groups compared with the Si-NC groups regardless of the addition of DMSO or 3-MA, and autophagy was decreased in both the *Si-PTK6* groups and the *Si-NC* group supplemented with 3-MA compared with the *Si-PTK6* groups and the Si-NC group supplemented with DMSO. As shown in Fig. 4C–G, C’–G’ and S7C–G, in SP6.5, MUM-2B and C918 cells, the proliferation, migration, and invasion abilities of the *Si-PTK6* groups were decreased compared with those of the *Si-NC* group regardless of the addition of DMSO or 3-MA. Moreover, the proliferation, migration, and invasion abilities of the *Si-PTK6* groups and the *Si-NC* group with 3-MA addition were increased compared with those of the *Si-PTK6* groups and the *Si-NC* group with DMSO addition. In contrast, we added rapamycin or DMSO to PTK6-overexpressing cells, as shown in Fig. 4H, I, H’, I’ and S7H, I, and autophagy was reduced in the LV-PTK6 group compared with the LV-NC group regardless of the DMSO or rapamycin treatment status; in addition, autophagy was enhanced in both the LV-PTK6 and LV-NC groups with rapamycin addition compared with the LV-PTK6 and LV-NC groups with DMSO addition in SP6.5, MUM-2B and C918 cells. As shown in Fig. 4J–N, J’–N’ and S7J–N, in SP6.5, MUM-2B and C918 cells, the proliferation, migration, and invasion abilities of the LV-PTK6 group were increased compared with those of the LV-NC group regardless of the DMSO or rapamycin treatment status. Moreover, the proliferation, migration, and invasion abilities of the LV-PTK6 group and the LV-NC group treated with rapamycin were decreased compared with those of the LV-PTK6 group and the LV-NC group treated with DMSO. Based on the above data, we concluded that PTK6 promotes UM tumorigenesis by inhibiting autophagy.

### SOCS3 binds to PTK6 and inhibits PTK6 expression

**Fig. 5:**
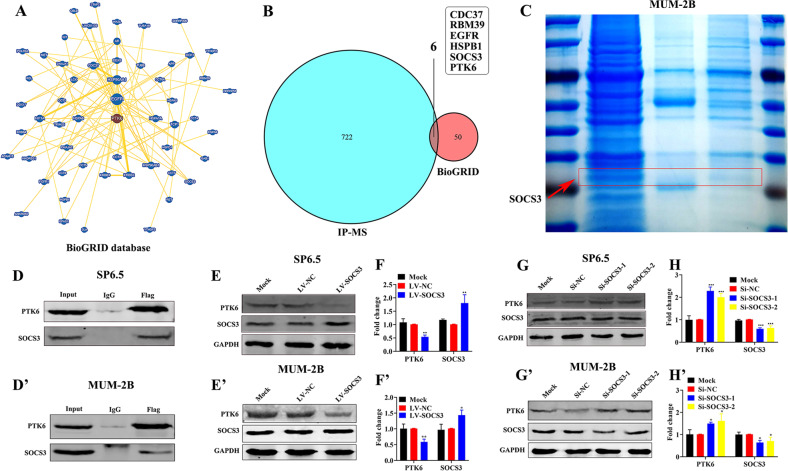
SOCS3 binds to PTK6 and inhibits PTK6 expression. **A** Network diagram of proteins related to PTK6 from the BioGRID database. **B** The Venn diagram of IP-MS and BioGRID. **C** The results of the SDS-PAGE visualized by Coomassie Brilliant Blue staining after immunoprecipitating. **D**, **D’** Coimmunoprecipitation analysis of PTK6 and SOCS3 in UM cells. **E**, **F** and **E’**, **F’** Western blotting analysis of SOCS3 and PTK6 in UM cells overexpressing SOCS3. **G**, **H** and **G’**, **H’** Western blotting analysis of SOCS3 and PTK6 in UM cells with SOCS3 knockdown. The above data were analyzed using one-way ANOVA with Bonferroni. (**D**–**H**: SP6.5 cell; **D**’–**H**’: MUM-2B cell; data were presented as the mean ± SD; *n* = 3; *p < 0.05, **p < 0.01, ***p < 0.001).

PTK6 is a tyrosine kinase, which consists of three protein domains (a classic src homology 3 (SH3) domains, a src homology 2 (SH2) domain, and a tyrosine kinase (SH1) domain). The protein domains of PTK6 are usually involved in protein-protein interactions [21]. Firstly, we obtained 55 proteins that might bind to PTK6 by the BioGRID database (Fig. 5A). Then, 727 proteins possibly binding to PTK6 were found by the technology of Immunoprecipitation Mass Spectrometry (IP-MS). We combined the results of the BioGRID database and IP-MS, and further found five common proteins (CDC37, RBM39, EGFR, HSPB1, and SOCS3) (Fig. 5B). Finally, we determined that PTK6 might bind to SOCS3 by the SDS-PAGE visualized by Coomassie Brilliant Blue staining (Fig. 5C). As shown in Fig. 5D, D’ and S8A, the PTK6 protein pulled down the SOCS3 protein. We verified that PTK6 is bound to SOCS3 by immunoprecipitation experiments. Meanwhile, we also demonstrated that the endogenic PTK6 can bind to SOCS3 (Fig. S16). It has been reported that SOCS3 can inhibit the expression of PTK6 protein [33–35]. Thus, we would like to verify whether PTK6 was a downstream target protein of SOCS3 in UM cells. Interestingly, in SP6.5, MUM-2B, and C918 cells, the expression of PTK6 was increased after knocking down SOCS3 (Fig. 5G, H, G’, H’ and S8B, C), while the expression of PTK6 was decreased after overexpressing SOCS3 (Fig. 5E, F, E’, F’ and S8D, E). This indicated that SOCS3 could inhibit the expression of PTK6 in UM.

### SOCS3 inhibits the proliferation, migration, and invasion of UM cells in vitro and in vivo

**Fig. 6:**
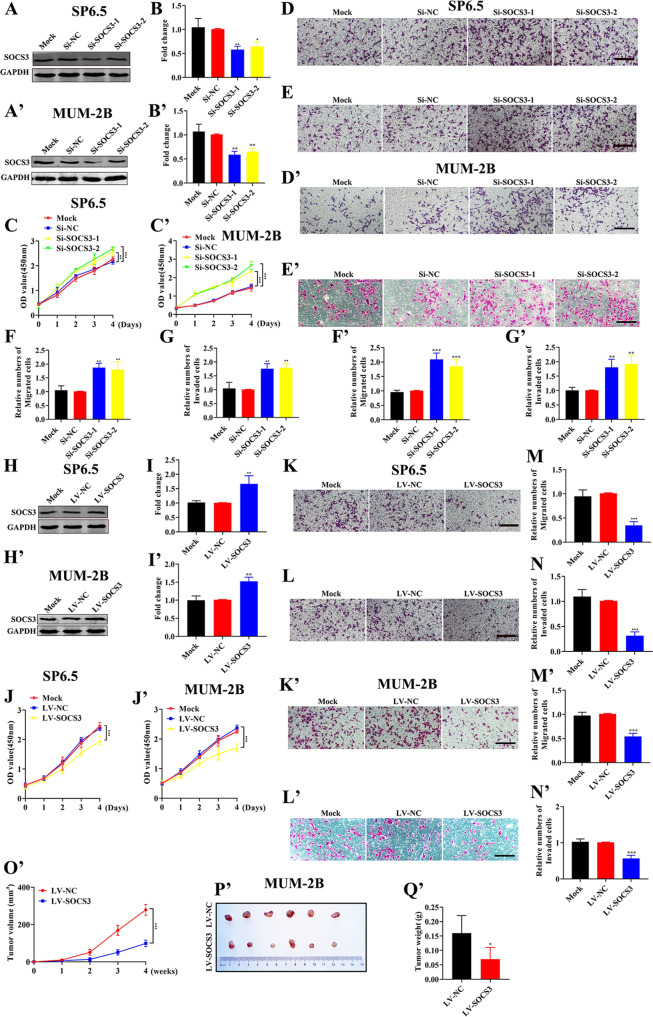
SOCS3 inhibits the proliferation, migration, and invasion of UM cells in vitro and in vivo. **A**, **B** and **A’**, **B’** Western blotting analysis of SOCS3 in UM cells with SOCS3 knockdown. The data were analyzed using one-way ANOVA with Bonferroni. **C**–**G** and **C’**–**G’** Analysis of the proliferation, migration, and invasion of UM cells with SOCS3 knockdown. **C** and **C’** were analyzed using two-way ANOVA with Bonferroni. **D**–**G** and **D’**–**G’** were analyzed using one-way ANOVA with Bonferroni. **H**, **I** and **H’**, **I’** Western blotting analysis of SOCS3 in UM cells overexpressing SOCS3. The data were analyzed using one-way ANOVA with Bonferroni. **J**–**N** and **J’**–**N** Proliferation, migration, and invasion analyses of UM cells overexpressing SOCS3. **J** and **J’** were analyzed using two-way ANOVA with Bonferroni. **K**–**N** and **K**’–**N**’ were analyzed using one-way ANOVA with Bonferroni. **O’** The volume of tumors formed in the LV-NC group and LV-SOCS3 group. The data were analyzed using two-way ANOVA with Bonferroni. **P’** Photographic images of tumors from the LV-NC group and LV-SOCS3 group. **Q’** The weight of tumors formed in the LV-NC group and LV-SOCS3 group. The data were analyzed using Student’s *t*-test. (**A**–**N**: SP6.5 cell; **A**’–**Q**’: MUM-2B cell; scale bar: 100 µm; data were presented as the mean ± SD; *n* = 3–6; ns no significant difference, *p < 0.05, **p < 0.01, ***p < 0.001).

Then, we further explored the function of *SOCS3* in UM. First, we detected the differential expression of SOCS3 in uveal melanocyte cells and UM cells. As shown in Fig. S9A–D, the expression of SOCS3 in UM cells was decreased compared with that in U-94 cells. Second, we knocked down and overexpressed *SOCS3* in UM. As shown in Fig. 6A, B, A’, B’, H, I, H’, I’ and S10A, B, H, I, SOCS3 was successfully knocked down and overexpressed in both SP6.5, MUM-2B, and C918 cell lines. We further examined the proliferation, migration, and invasion of UM cells. As shown in Fig. 6C–G, C’–G’ and S10C–G, in SP6.5, MUM-2B, and C918 cells, the proliferation, migration, and invasion abilities of the *Si-SOCS3* groups were stronger than those of the *Si-NC* group. In contrast, as shown in Fig. 6J–N, J’–N’ and S10J–N, the proliferation, migration, and invasion abilities of UM cells overexpressing SOCS3 (LV-SOCS3) were weaker than those of the control group (LV-NC). Finally, LV-SOCS3 and LV-NC cells were injected subcutaneously into nude mice, and we examined the expression of SOCS3 protein in the two groups of tumors (S14B, E). As shown in Fig. 6O’–Q’ and S10O–Q, the volume and weight of tumors in the LV-SOCS3 group were significantly smaller than those in the LV-NC group. The above data indicated that SOCS3 inhibits UM tumorigenesis in vivo and in vitro.

### Overexpressing SOCS3 can partially attenuate the PTK6-induced promotion of UM cell proliferation, migration, and invasion in vitro and in vivo

**Fig. 7:**
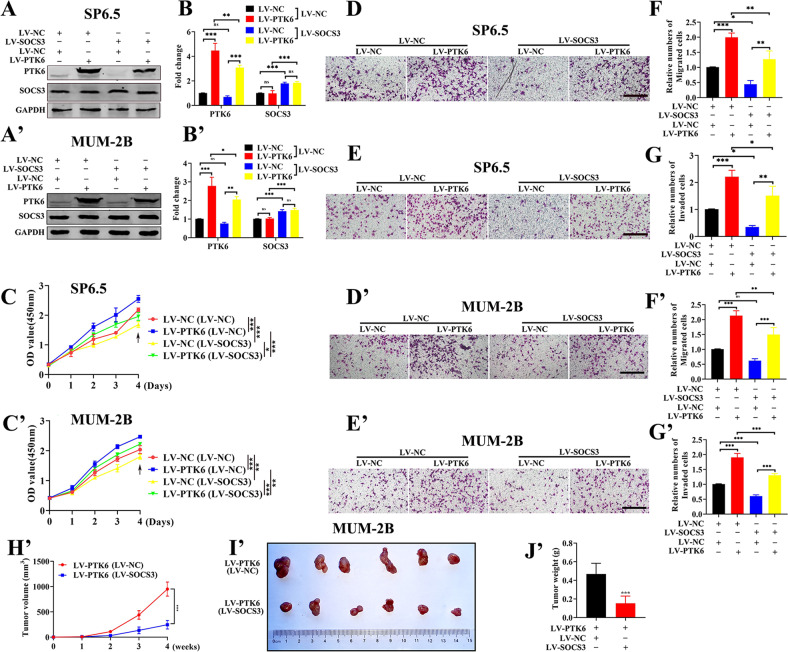
Overexpressing SOCS3 can partially attenuate the PTK6-induced promotion of UM cell proliferation, migration, and invasion in vitro and in vivo. **A**, **B** and **A’**, **B’** Western blotting analysis of SOCS3 and PTK6 in the LV-NC group or LV-PTK6 group supplemented with LV-NC and LV-SOCS3. **C**–**G** and **C’**–**G’** Proliferation, migration, and invasion analysis of the LV-NC group or LV-PTK6 group treated with LV-NC and LV-SOCS3. The above data were analyzed using two-way ANOVA with Bonferroni. **H’** The volume of tumors formed in the LV-PTK6/LV-NC group and LV-PTK6/LV-SOCS3 group. The data were analyzed using two-way ANOVA with Bonferroni. **I’** Photographs of tumors from the LV-PTK6/LV-NC group and LV-PTK6/LV-SOCS3 group. **J’** The weight of tumors formed in the LV-PTK6/LV-NC group and LV-PTK6/LV-SOCS3 group. The data were analyzed using Student’s *t*-test. (**A**–**G**: SP6.5 cell; **A’**–**J’**: MUM-2B cell; scale bar: 100 µm; data were presented as the mean ± SD; *n* = 3–6; ns no significant difference, *p < 0.05, **p < 0.01, ***p < 0.001).

Based on the above results, we explored whether SOCS3 can attenuate the proliferation, migration, and invasion of UM promoted by PTK6. We transfected LV-NC or LV-SOCS3 lentiviral overexpression plasmid into LV-NC and LV-PTK6 cell lines and then detected the proliferation, migration, and invasion of UM cells. As shown in Fig. 7A, B, A’, B’ and S11A, B, four cell lines named LV-NC/LV-NC, LV-PTK6/LV-NC, LV-NC/LV-SOCS3, and LV-PTK6/LV-SOCS3 were successfully constructed. As shown in Fig. 7C–G, C’–G’ and S11C–G, in SP6.5, MUM-2B and C918 cells, the proliferation, migration, and invasion abilities of the LV-PTK6 group were increased compared with those in the LV-NC group regardless of whether LV-NC or LV-SOCS3 plasmids were applied, and the proliferation, migration, and invasion abilities of the LV-PTK6 group and the LV-NC group applied LV-SOCS3 plasmids were decreased compared with those of the LV-PTK6 group and the LV-NC group applied LV-NC plasmids. Finally, LV-PTK6/LV-NC and LV-PTK6/LV-SOCS3 cells were injected subcutaneously into nude mice, and we examined the expression of PTK6 and SOCS3 proteins in the two groups of tumors (S14C, F). As shown in Fig. 7H’–J’ and S11H–J, the volume and weight of tumors in the LV-PTK6/LV-SOCS3 group were smaller than those in the LV-PTK6/LV-NC group. The above data demonstrated that SOCS3 can attenuate UM tumorigenesis induced by PTK6 in vivo and in vitro.

We further examined whether autophagy was changed in four cell lines (LV-NC/LV-NC, LV-PTK6/LV-NC, LV-NC/LV-SOCS3, and LV-PTK6/LV-SOCS3). Interestingly, we found that SOCS3 can partially rescue the PTK6-inhibited autophagy (Fig. S12). This indicated that SOCS3 was involved in the autophagy of PTK6 in uveal melanoma. SOCS3 plays an important role in a variety of diseases, including tumors, and resveratrol has been found to increase SOCS3 expression, which offers the possibility of pharmacological intervention for PTK6 [36, 37]. As shown in Fig. S15A, C, resveratrol can upregulate the expression of SOCS3 and downregulate the expression of PTK6. Meanwhile, resveratrol can inhibit the proliferation, migration, and invasion of UM cells (Fig. S15B, D–J).

### PTK6 promotes mTOR phosphorylation

**Fig. 8:**
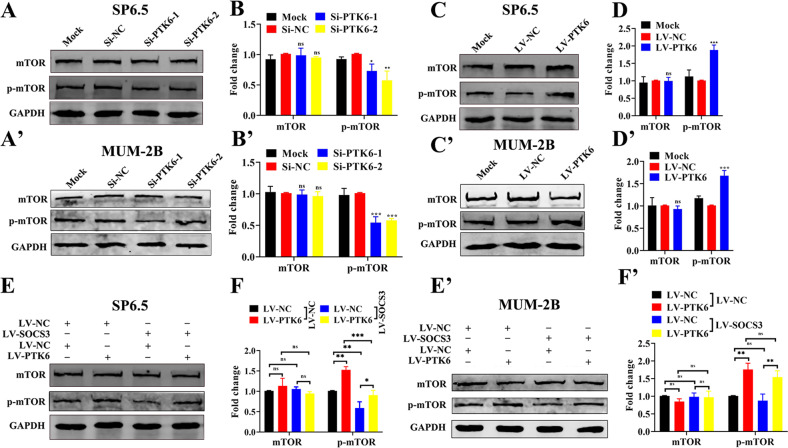
PTK6 promotes mTOR phosphorylation. **A**, **B and A’**, **B’** Western blotting analysis of mTOR and p-mTOR in UM cells with PTK6 knockdown. **C**, **D** and **C’**, **D’** Western blotting analysis of mTOR and p-mTOR in UM cells overexpressing PTK6. The above data were analyzed using one-way ANOVA with Bonferroni. **E**, **F** and **E’**, **F’** Western blotting analysis of mTOR and p-mTOR in the four cell lines: LV-NC/LV-NC, LV-PTK6/LV-NC, LV-NC/LV-SOCS3, and LV-PTK6/LV-SOCS3. The data were analyzed using two-way ANOVA with Bonferroni. (**A**–**F**: SP6.5 cell; **A**’–**F**’: MUM-2B cell; data were presented as the mean ± SD; *n* = 3; ns no significant difference, *p < 0.05, **p < 0.01, ***p < 0.001).

Finally, we explored whether PTK6 can induce the phosphorylation of downstream proteins. It is well known that SOCS3 is closely related to the mTOR signaling pathway, so we further assessed whether PTK6 affects the expression of mTOR protein. We detected the changes in mTOR and p-mTOR protein expression after knocking down or overexpressing PTK6 in UM cells. In SP6.5, MUM-2B, and C918 cells, the expression of mTOR protein was not significantly changed in the *Si-PTK6* groups compared with the *Si-NC* groups, but the expression of the p-mTOR protein was decreased (Fig. 8A, B, A’, B’ and S13A, B); the expression of the mTOR protein was not significantly changed in the LV-PTK6 group compared with the LV-NC group, but the expression of the p-mTOR protein was increased (Fig. 8C, D, C’, D’ and S13C, D). We also detected the expression of mTOR and p-mTOR proteins in the four cell lines LV-NC/LV-NC, LV-PTK6/LV-NC, LV-NC/LV-SOCS3, and LV-PTK6/LV-SOCS3. As shown in Fig. 8E, F, E’, F’ and S13E, F, SOCS3 attenuated the phosphorylation of the mTOR protein induced by PTK6. The above data indicated that PTK6 promotes the phosphorylation of mTOR protein.

**Fig. 9:**
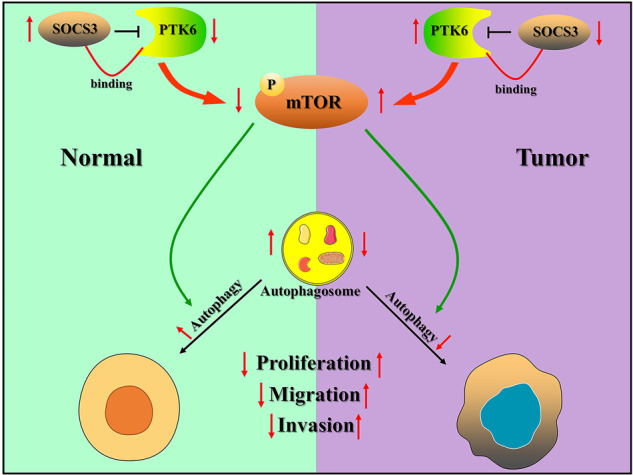
Proposed schematic illustration of the PTK6 mechanism: PTK6 inhibits autophagy to promote UM tumorigenesis by binding to SOCS3 and regulating mTOR phosphorylation.

Taken together, our data demonstrate that PTK6 can bind to SOCS3, SOCS3 can inhibit the expression of PTK6, and PTK6 promotes the phosphorylation of the mTOR protein. This then promotes UM tumorigenesis by inhibiting autophagy (Fig. 9).

## Discussion

This study reveals that PTK6 can promote the development of UM by inhibiting autophagy. SOCS3, whose function is to suppress the growth of UM, can bind to PTK6, and the phosphorylation of the mTOR protein can be induced by PTK6. Importantly, SOCS3 can inhibit the expression of PTK6, while overexpression of SOCS3 can attenuate UM tumorigenesis caused by PTK6. We clarified the relationship between PTK6 and SOCS3, as well as the interaction between PTK6 and autophagy, which provides a theoretical basis for the targeting of these molecules in UM treatment.

Previous studies have indicated that the abnormal expression of PTK6 is related to a variety of malignant tumors, including breast cancer, hepatocellular carcinoma, esophageal squamous cell carcinoma, prostate cancer, and colorectal cancer [21, 24, 38–40]. However, the role of PTK6 in different tumors is still controversial, which might be due to PTK6 activating different signaling pathways. PTK6 can promote the tumorigenesis of breast cancer by interacting with the EGFR pathway [22]. Sam68, as a substrate of PTK6, colocalizes with PTK6 to promote the growth of prostate tumor cells [41]. PTK6 promotes STAT3 activation in UVB-induced skin tumors [42]. In contrast, PTK6 plays an inhibitory role in the development of colorectal cancer and nasopharyngeal carcinoma. PTK6 plays a tumor suppressor role in colorectal cancer cells by antagonizing the epithelial-mesenchymal transition (EMT) pathway [43]. Downregulated expression of PTK6 correlates with the poor survival of esophageal squamous cell carcinoma [44]. Our study first showed that PTK6 promotes the proliferation, migration, and invasion of UM cells (Fig. 2), which is also a supplement to previous research on the function of PTK6 in tumors. Furthermore, we illustrate that PTK6 promotes the growth of UM by inhibiting autophagy (Fig. 3). The application of autophagy agonizts and inhibitors indicated that autophagy activation can inhibit UM tumorigenesis (Fig. 4), which is also consistent with the results of previous studies.

SOCS3 is a negative regulator of cytokine signal transduction that can bind to cellular surface-specific receptors [45], regulate various signaling pathways, and transfer extracellular signals to the intracellular space [46]. In our study, SOCS3 bound to PTK6 and suppressed the expression of PTK6, and SOCS3 rescued the PTK6-induced oncogenicity of UM (Fig. 7). Previous literature has shown that dysfunction of SOCS3 might cause a variety of diseases, including immunological diseases, neurodegenerative diseases and tumors [45]. For example, SOCS3 can suppress the induction and development of rheumatoid arthritis [47], and SOCS3 is also closely related to the progression of asthma [48]. In retinal studies, the deletion of SOCS3 can promote optic nerve regeneration [49], and SOCS3 is involved in the development of age-related retinal degeneration by mediating microglial activity [50]. For tumors, the function of SOCS3 depends on the tumor type. In general, it is believed that SOCS3 can inhibit tumors, including breast cancer and ovarian cancer. However, SOCS3 may have a stimulative effect on tumors, including gastric cancer and renal cell carcinoma [51]. Our data indicated that SOCS3 inhibited the proliferation, migration, and invasion of UM (Fig. 6). This reminds us that more studies are needed to investigate the function of SOCS3 in specific tumors. An increasing number of studies have shown that, mechanistically, SOCS3 regulates the JAK/STAT3 signaling pathway in various diseases [52–54]. SOCS3 can also suppress the NF-κB signaling pathway [48], and the expression of some cytokines, such as IL-2, IL-6, IL-12, and TNFs, in the inflammatory pathway can be induced by SOCS3 [45, 55, 56]. Importantly, SOCS3 can inhibit the mTOR pathway [57]. mTOR is a serine and threonine protein kinase that plays a key role in cell growth, cellular energy metabolism, protein synthesis, and autophagy. Dysregulation of the mTOR signaling pathway is related to neurodegeneration, aging, and the development of cancer and diabetes [58, 59]. Previous studies have shown that the mTOR signaling pathway might affect the progression of diseases by inhibiting autophagy [60, 61] and that the activation of mTOR might be caused by the phosphorylation of mTOR in tumors [62]. Our study demonstrates that PTK6 can suppress the autophagy pathway by promoting the phosphorylation of the mTOR protein in UM (Fig. 8). This indicates that mTOR might serve as a downstream effector of PTK6 in promoting UM tumorigenesis. Our findings are consistent with those of previous reports regarding the role of the mTOR pathway in tumors, which may have implications for the treatment of UM patients.

To complement our experiments, the functions of PTK6 and SOCS3 were also explored in normal cells, including U-94 and ARPE-19 cells. The expression of PTK6 was decreased in U-94 cells (Fig. S2), while the expression of SOCS3 was increased (Fig. S9). Interestingly, we found that PTK6 could also inhibit autophagy and promote the phosphorylation of the mTOR protein (Fig. S17) and that SOCS3 could bind to PTK6 and inhibit the expression of PTK6 in ARPE-19 cells (Fig. S18). The above data indicate that PTK6 inhibits autophagy and that the interaction between PTK6 and SOCS3 is a common phenomenon. According to previous studies, PTK6 can be used as an autophagy-related gene to evaluate the prognosis of patients with tumors, including glioma, prostate cancer, and pancreatic cancer [63–65]. In normal tissues, PTK6 can affect cellular differentiation, apoptosis, and cell cycle progression [20]. This suggests that PTK6 not only plays a tumor-promoting role by inhibiting autophagy in UM but might also play a role by suppressing the autophagy pathway in other disease models.

Currently, an effective understanding of genetic complexity contributes to the individualized treatment of UM. As mentioned in the introduction, although some mutant genes have been shown to be related to the occurrence and development of UM [1, 6], it is still unclear whether there are any new pathogenic genes that inhibit or promote the progression of UM. Our work has indicated that PTK6 or SOCS3 can promote or inhibit UM tumorigenesis and that overexpression of SOCS3 can attenuate the proliferation, migration, and invasion of UM cells induced by PTK6 (Figs. 2, 6, 7). The interaction complex of PTK6 and SOCS3 is helpful for us to understand the molecular mechanism of UM and provides new evidence for the polygenic therapy of UM. However, there are some shortcomings in our work. The expression of PTK6 and SOCS3 was not validated in vivo due to the lack of UM tissue samples. Furthermore, we did not demonstrate the occurrence of PTK6-inhibited autophagy or the relationship between SOCS3 and PTK6 in vivo. However, we verified the relationship between PTK6 and SOCS3 in multiple cell lines, which helped us to further understand the pathogenesis of UM.

In summary, we found that PTK6 is overexpressed in UM, which promotes the formation of UM by inhibiting autophagy. We also found that overexpressing SOCS3 can partially rescue the PTK6-induced UM tumorigenesis. Mechanistically, PTK6 can bind to SOCS3, SOCS3 can downregulate the expression of PTK6, and PTK6 can upregulate the phosphorylation of the mTOR protein. Our work uncovers the critical functions of PTK6 and SOCS3 in UM and provides novel insight into the treatment of UM.

## Supplementary information

Supplementary Information

Original Data File

Checklist
