# Supplementary material for: PTK6 inhibits autophagy to promote uveal melanoma tumorigenesis by binding to SOCS3 and regulating mTOR phosphorylation
Source: Cell Death Dis. 2023 Jan 23;14(1):55. doi: 10.1038/s41419-023-05590-w (PMC9870980; doi:10.1038/s41419-023-05590-w)
Supplement: Supplementary file 1 — Supplementary Information [file 41419_2023_5590_MOESM1_ESM.docx]

**Supplementary information**


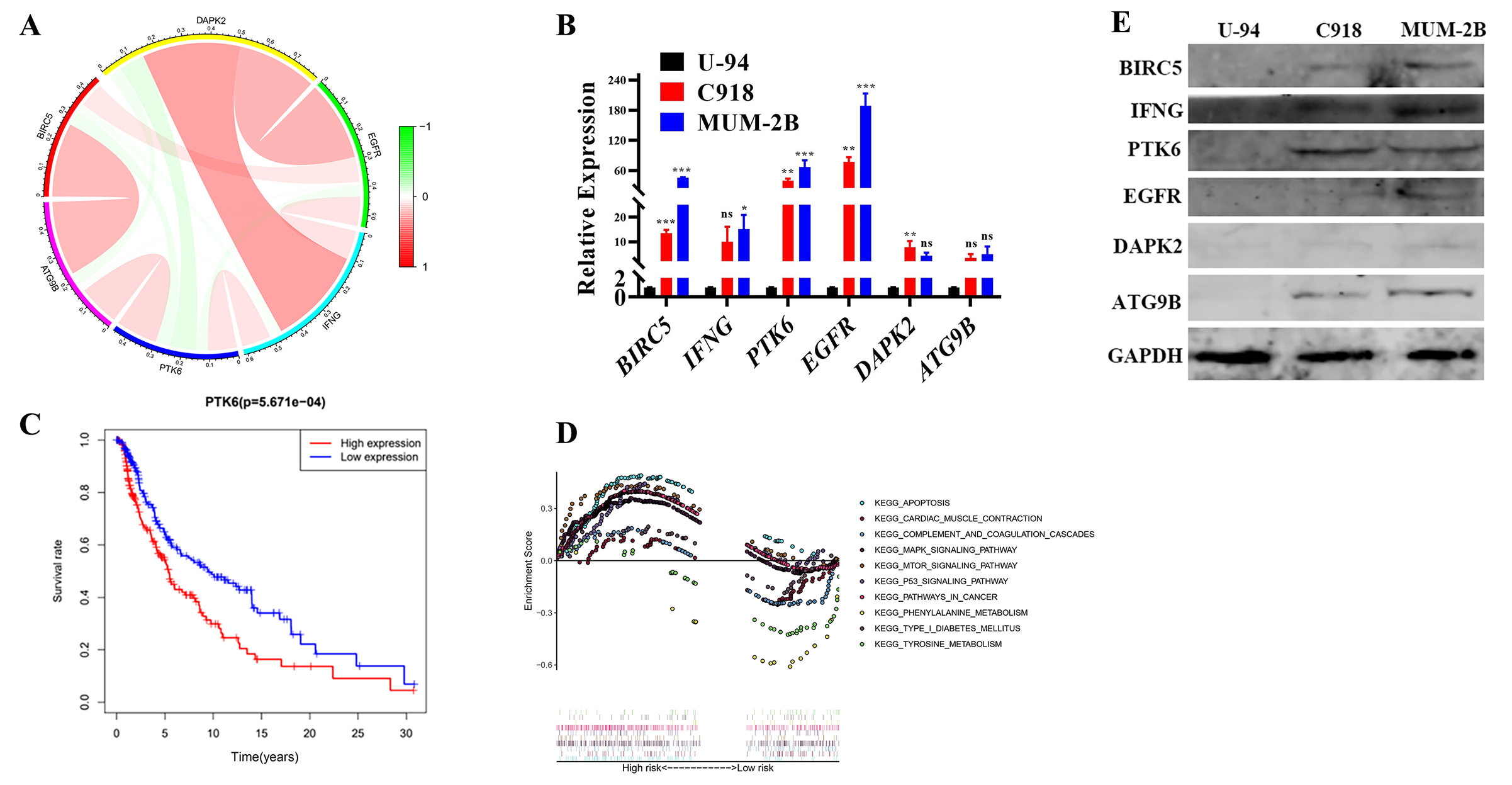


**Fig. S1 PTK6 expression is correlated with the survival of UM patients.**

**(A)** The correlation circle graph of 6 ARGs in UM. **(B)** The expression of 6 ARG mRNAs in U-94 and UM cells. **(C)** OS curves for melanoma patients in the *PTK6* high-expression group and the *PTK6* low-expression group. **(D)** GSEA of PTK6 in UM. **(E)** Western blotting analysis of BIRC5, IFNG, PTK6, EGFR, DAPK2 and ATG9B in U-94 and UM cells. (Data are presented as the mean ± SD; n = 3; ns: no significant difference, *p < 0.05, **p < 0.01, ***p < 0.001).


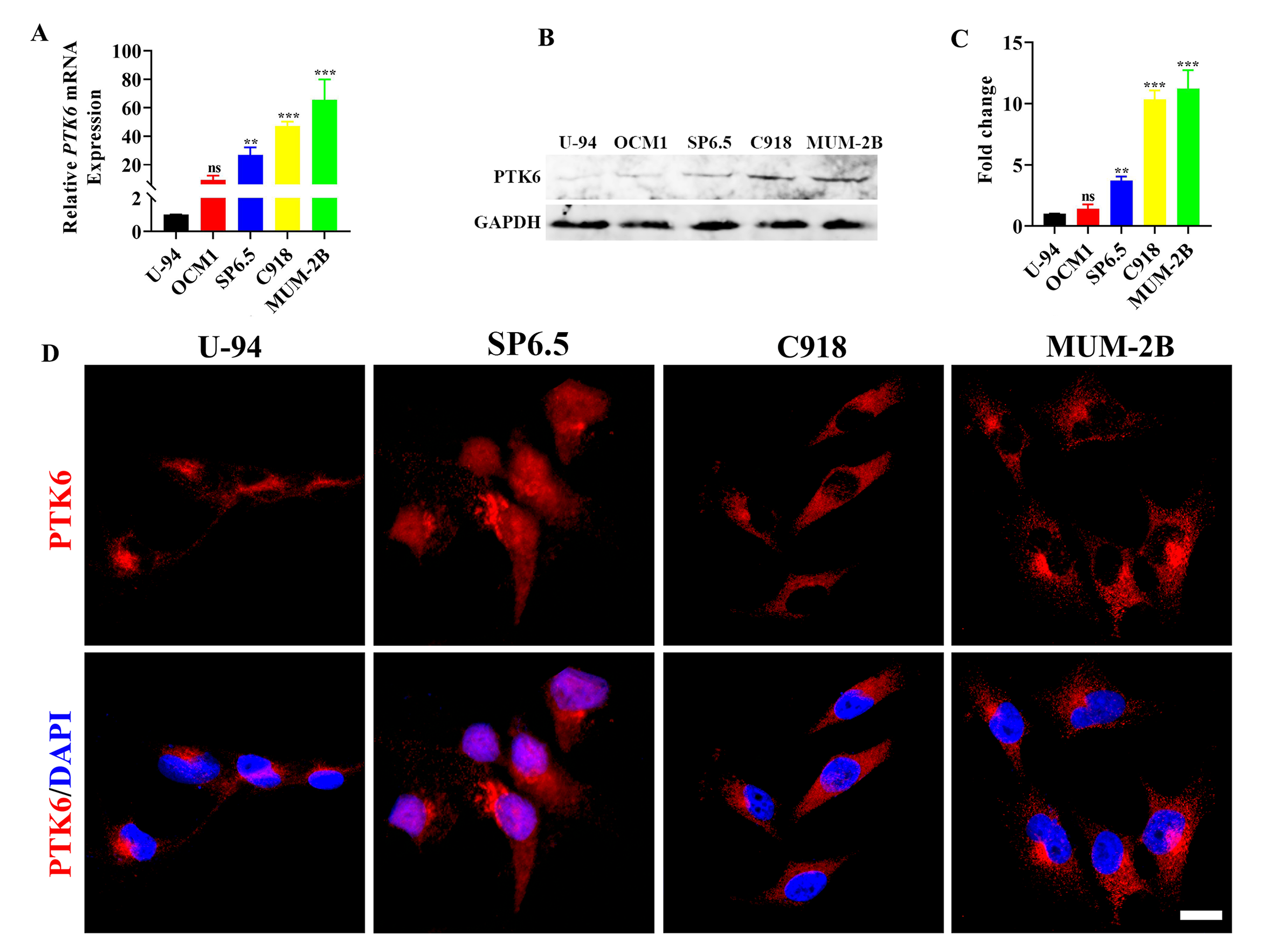


**Fig. S2 The expression of PTK6 in UM cells.**

**(A)** The expression of PTK6 mRNA in U-94 and UM cells. **(B and C)** Western blotting analysis of PTK6 in U-94 and UM cells. The above datas were analyzed using one-way ANOVA with Bonferroni. **(D)** Immunofluorescence analysis of PTK6 in U-94 and UM cells. (Scale bar: 50 µm; data are presented as the mean ± SD; n = 3; ns: no significant difference, **p < 0.01, ***p < 0.001).


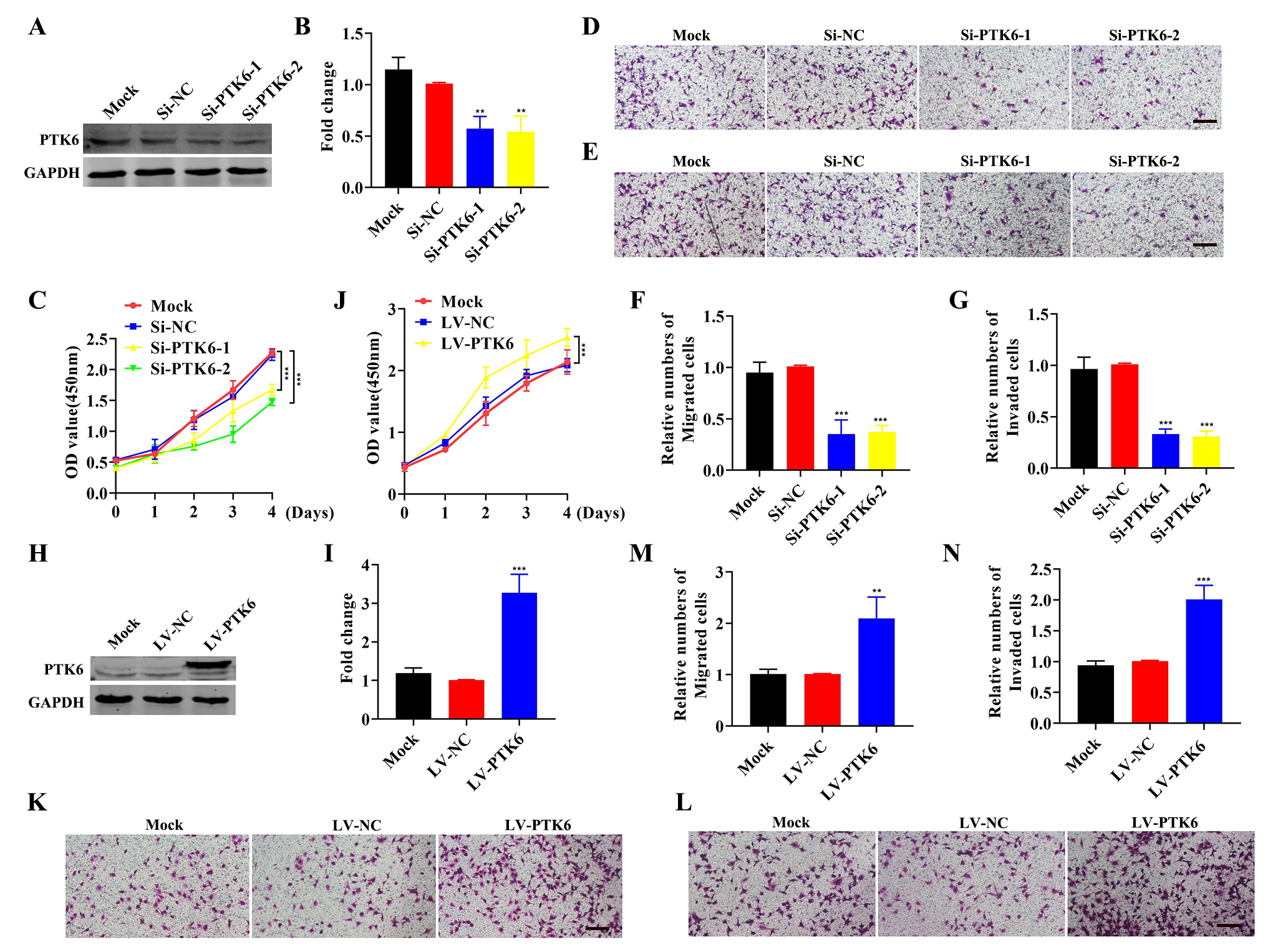


**Fig. S3 PTK6 promotes the proliferation, migration, and invasion of** **skin melanoma cell**

**(A-B)** Western blotting analysis of PTK6 in skin melanoma cell with PTK6 knockdown. The above datas were analyzed using one-way ANOVA with Bonferroni. **(C-G)** Analysis of the proliferation, migration, and invasion of skin melanoma cell with PTK6 knockdown. C was analyzed using two-way ANOVA with Bonferroni. D-G was analyzed using one-way ANOVA with Bonferroni. **(H-I)** Western blotting analysis of PTK6 in skin melanoma cell overexpressing PTK6. The above data were analyzed using one-way ANOVA with Bonferroni. **(J-N)** Analysis of the proliferation, migration, and invasion of skin melanoma cell overexpressing PTK6. J was analyzed using two-way ANOVA with Bonferroni. K-N was analyzed using one-way ANOVA with Bonferroni. (A-N: A375 cell; scale bar: 100 µm; data are presented as the mean ± SD; n = 3; *p < 0.05, **p < 0.01, ***p < 0.001).


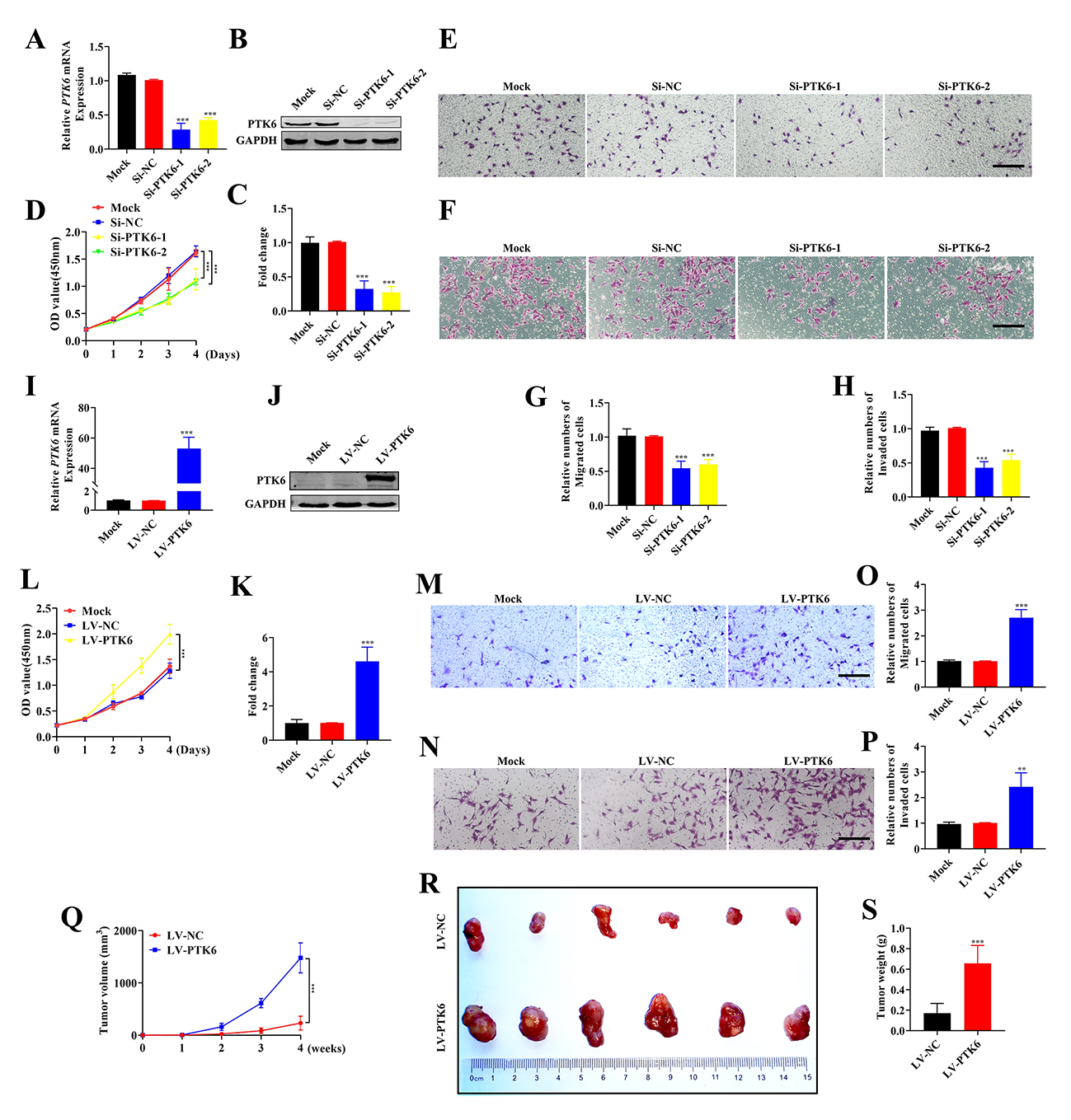


**Fig. S4 PTK6 promotes the proliferation, migration, and invasion of UM cell in vitro and in vivo.**

**(A)** The expression of PTK6 mRNA in UM cell with PTK6 knockdown. **(B-C)** Western blotting analysis of PTK6 in UM cell with PTK6 knockdown. The above datas were analyzed using one-way ANOVA with Bonferroni. **(D-H)** Analysis of the proliferation, migration, and invasion of UM cell with PTK6 knockdown. D was analyzed using two-way ANOVA with Bonferroni. E-H was analyzed using one-way ANOVA with Bonferroni. **(I)** The expression of PTK6 mRNA in UM cell overexpressing PTK6. **(J-K)** Western blotting analysis of PTK6 in UM cell overexpressing PTK6. The above data were analyzed using one-way ANOVA with Bonferroni. **(L-P)** Analysis of the proliferation, migration, and invasion of UM cell overexpressing PTK6. L was analyzed using two-way ANOVA with Bonferroni. M-P were analyzed using one-way ANOVA with Bonferroni. **(Q)** The volume of tumors formed in the LV-NC group and LV-PTK6 group. The datas were analyzed using two-way ANOVA with Bonferroni. **(R)** Photographic images of tumors from the LV-NC group and LV-PTK6 group. **(S)** The weight of tumors formed in the LV-NC group and LV-PTK6 group. The datas were analyzed using Student’s t-test. (A-S: C918 cell; scale bar: 100 µm; data are presented as the mean ± SD; n = 3–6; *p < 0.05, **p < 0.01, ***p < 0.001).

**
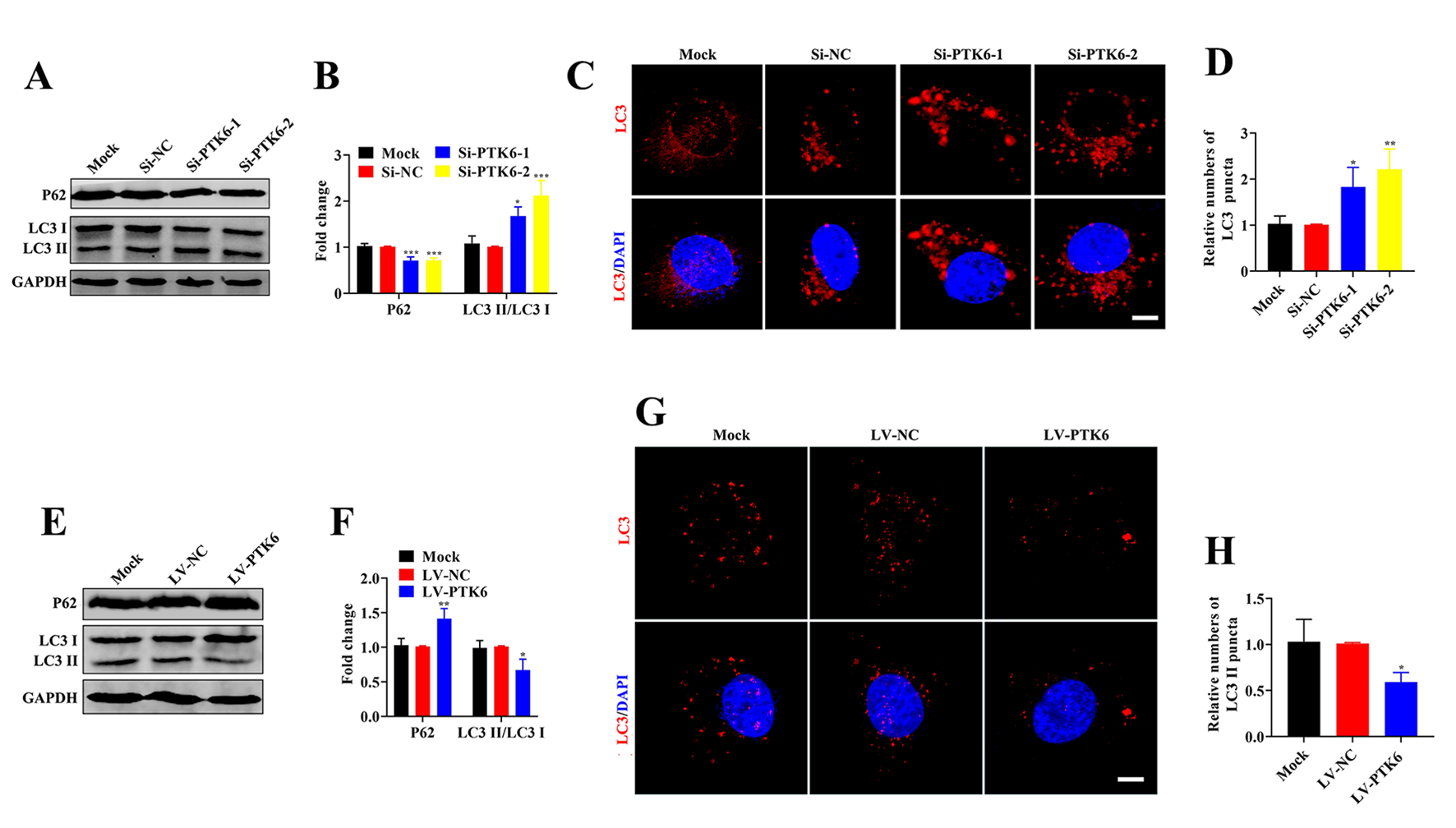
 Fig. S5** **PTK6** **inhibits autophagy in UM cell.**

**(A-B)** Western blotting analysis of LC3 and P62 in UM cell with PTK6 knockdown. **(C-D)** Immunofluorescence analysis of autophagosomes in UM cell with PTK6 knockdown. The above datas were analyzed using one-way ANOVA with Bonferroni. **(E-F)** Western blotting analysis of LC3 and P62 in UM cell overexpressing PTK6. **(G-H)** Immunofluorescence analysis of autophagosomes in UM cell overexpressing PTK6. The above datas were analyzed using one-way ANOVA with Bonferroni. (A-H: C918 cell; scale bar: 20 µm; data are presented as the mean ± SD; n=3; *p < 0.05, **p < 0.01, ***p < 0.001).


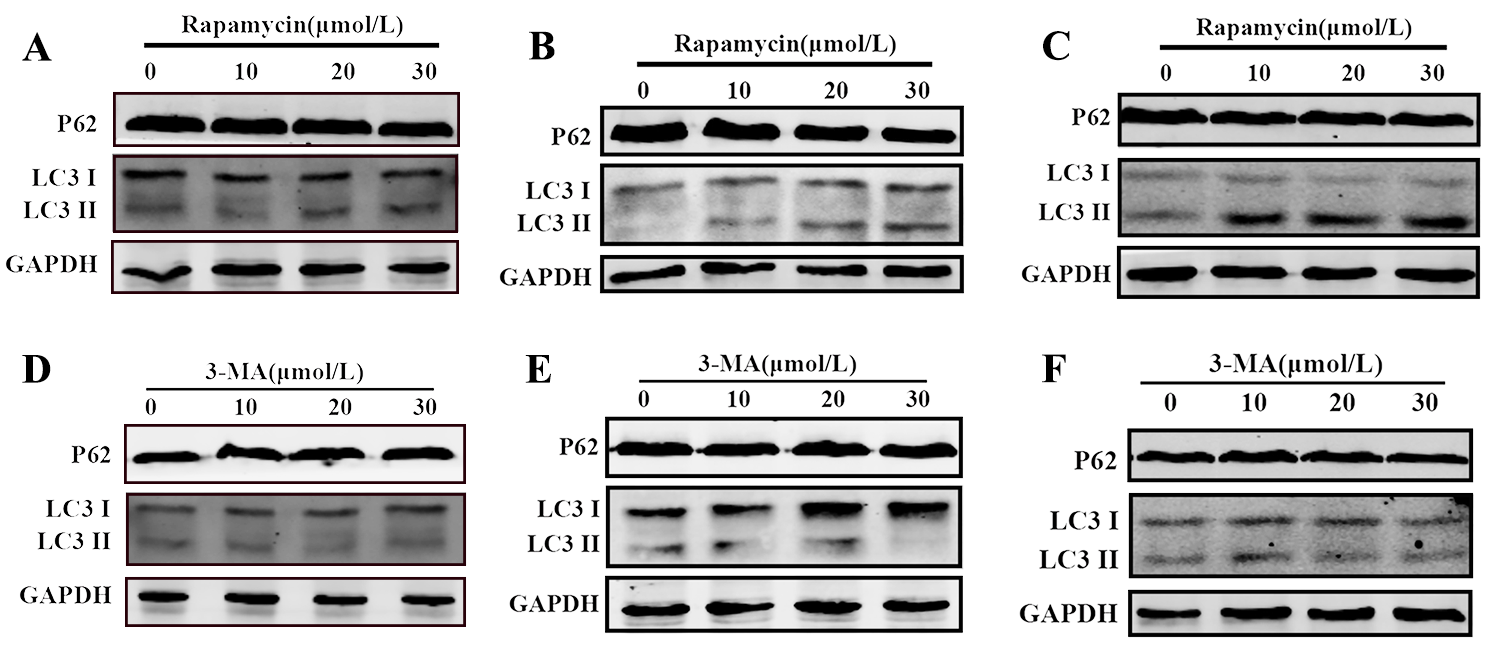


**Fig. S6 The suitable concentration of rapamycin and 3-MA in UM cells.**

**(A-C)** Western blotting of LC3 and P62 in UM cells treated with different concentrations of rapamycin. **(D-F)** Western blotting of LC3 and P62 in UM cells treated with different concentrations of 3-MA. (A and D: SP6.5 cell; B and E: C918 cell; C-F: MUM-2B cell).


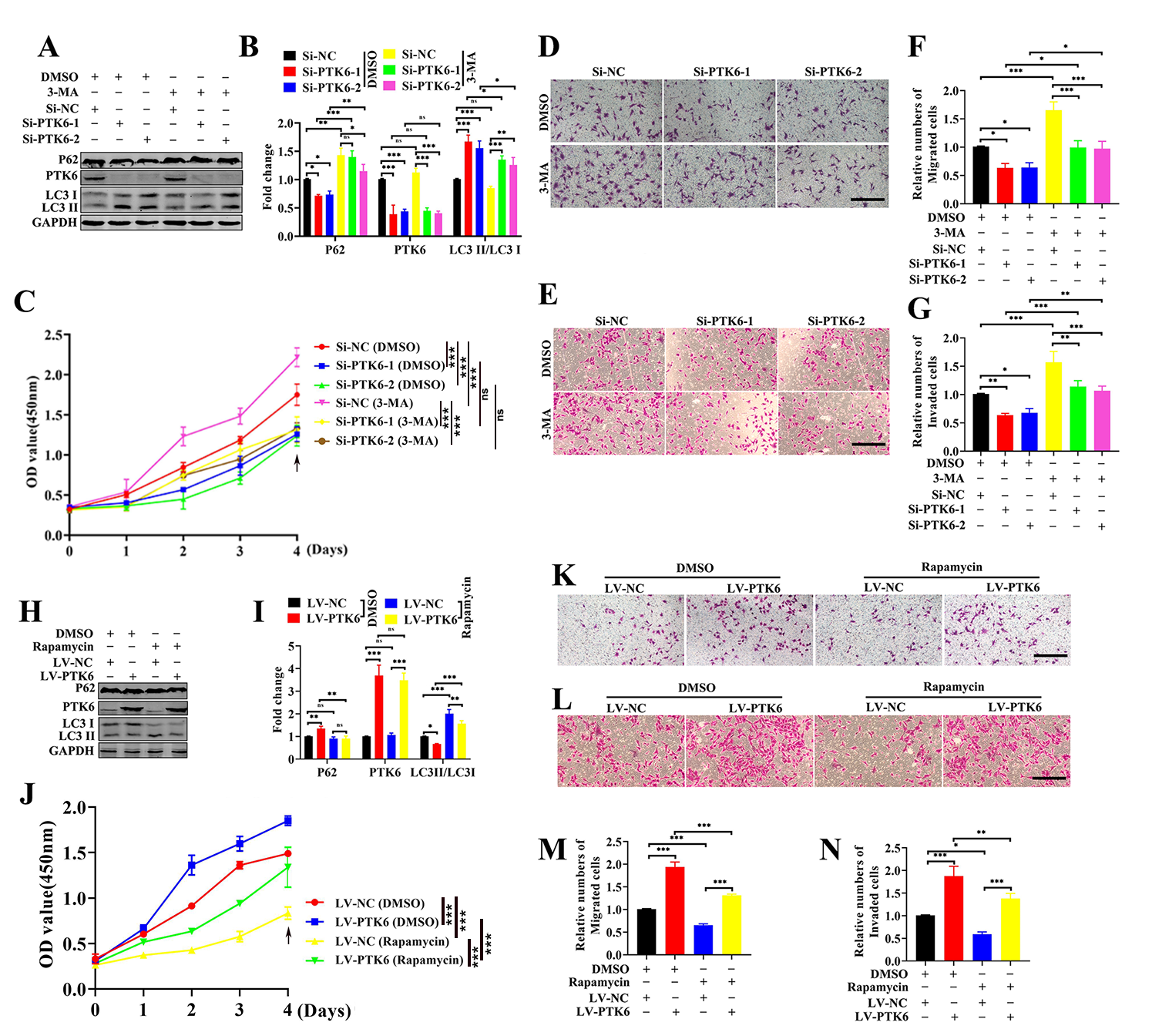


**Fig. S7 PTK6 promotes the proliferation, migration, and invasion of UM cell by inhibiting autophagy**.

**(A-B)** Western blotting analysis of LC3, PTK6, and P62 in the *Si-PTK6* groups and *Si-NC* group after treatment with DMSO or 3-MA. The datas were analyzed using two-way ANOVA with Bonferroni. **(C-G)** Proliferation, migration, and invasion analyses of the *Si-PTK6* groups and *Si-NC* group after treatment with DMSO or 3-MA. The datas were analyzed using two-way ANOVA with Bonferroni. **(H-I)** Western blotting analysis of LC3, PTK6, and P62 in the LV-NC group and LV-PTK6 group after treatment with DMSO or rapamycin. The datas were analyzed using two-way ANOVA with Bonferroni. **(J-N)** Proliferation, migration, and invasion analyses of the LV-NC group and LV-PTK6 group after treatment with DMSO or rapamycin. The datas were analyzed using two-way ANOVA with Bonferroni. (A-N: C918 cell; scale bar: 100 µm; data are presented as the mean ± SD; n = 3; ns: no significant difference, *p < 0.05, **p < 0.01, ***p < 0.001).


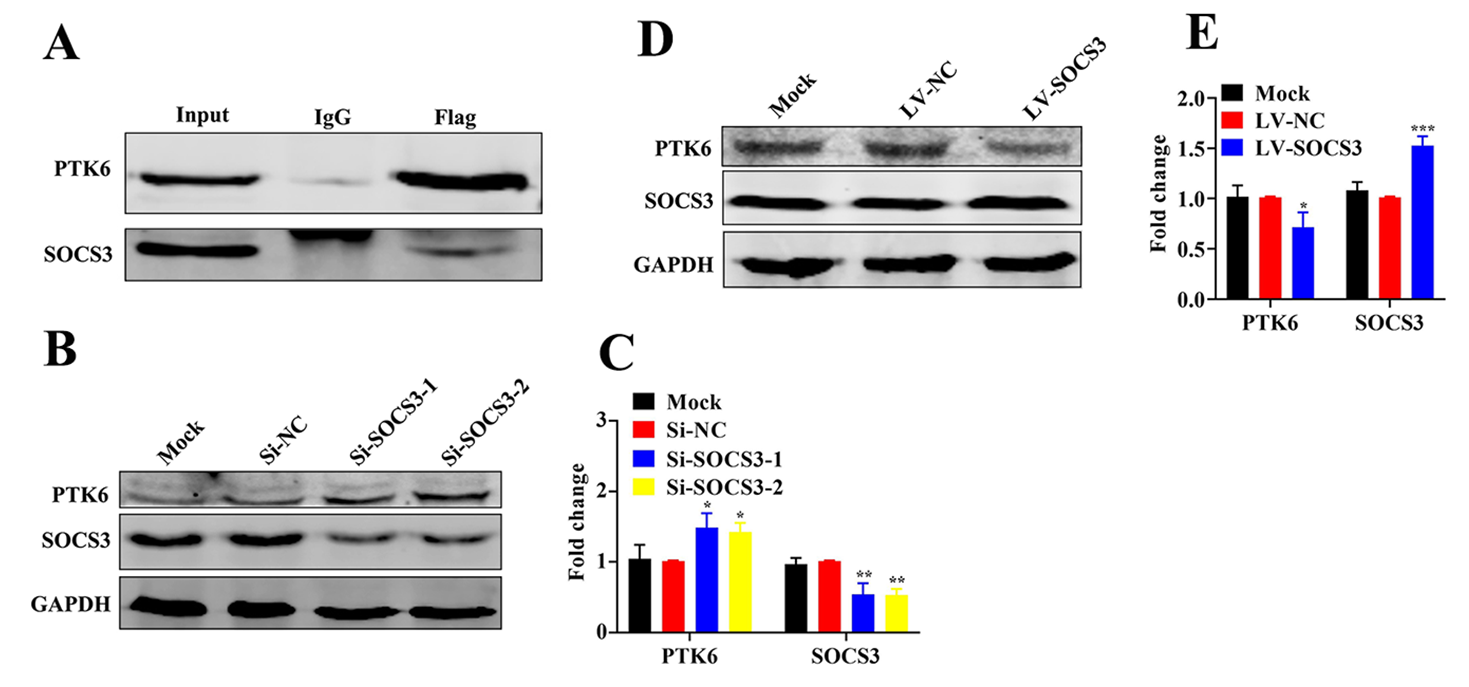


**Fig. S8 SOCS3 binds to PTK6 and inhibits PTK6 expression.**

**(A)** Coimmunoprecipitation analysis of PTK6 and SOCS3 in UM cell. **(B-C)** Western blotting analysis of SOCS3 and PTK6 in UM cell with SOCS3 knockdown. **(D-E)** Western blotting analysis of SOCS3 and PTK6 in UM cell overexpressing SOCS3. The above datas were analyzed using one-way ANOVA with Bonferroni. (A-E: C918 cell; data are presented as the mean ± SD; n = 3; *p < 0.05, **p < 0.01, ***p < 0.001).


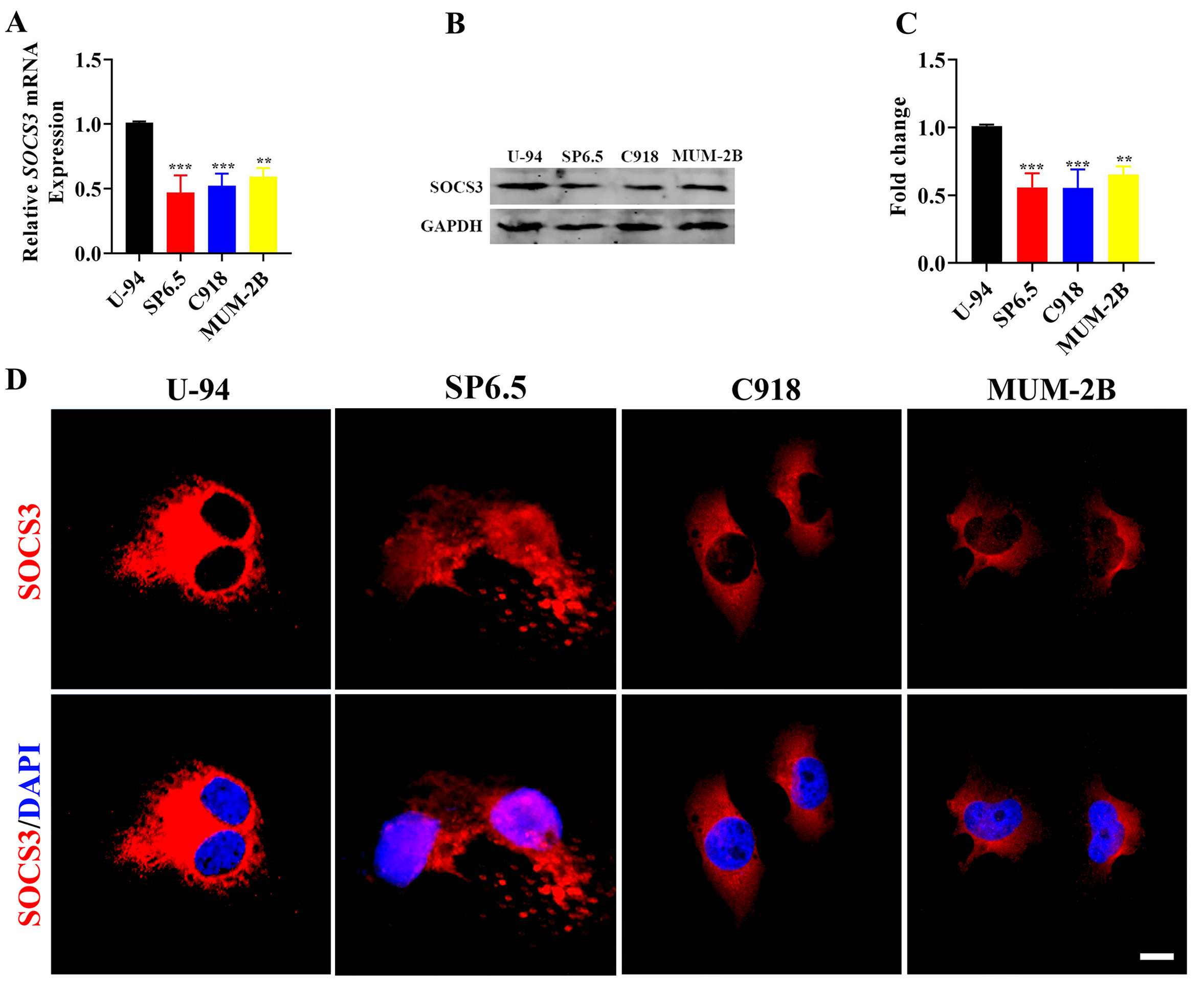


**Fig. S9 The expression of SOCS3 in UM cells.**

**(A)** The expression of SOCS3 mRNA in U-94 and UM cells. **(B and C)** Western blotting analysis of SOCS3 in U-94 and UM cells. The above datas were analyzed using one-way ANOVA with Bonferroni. **(D)** Immunofluorescence analysis of SOCS3 in U-94 and UM cells. (Scale bar: 50 µm; data are presented as the mean ± SD; n = 3; **p < 0.01, ***p < 0.001).


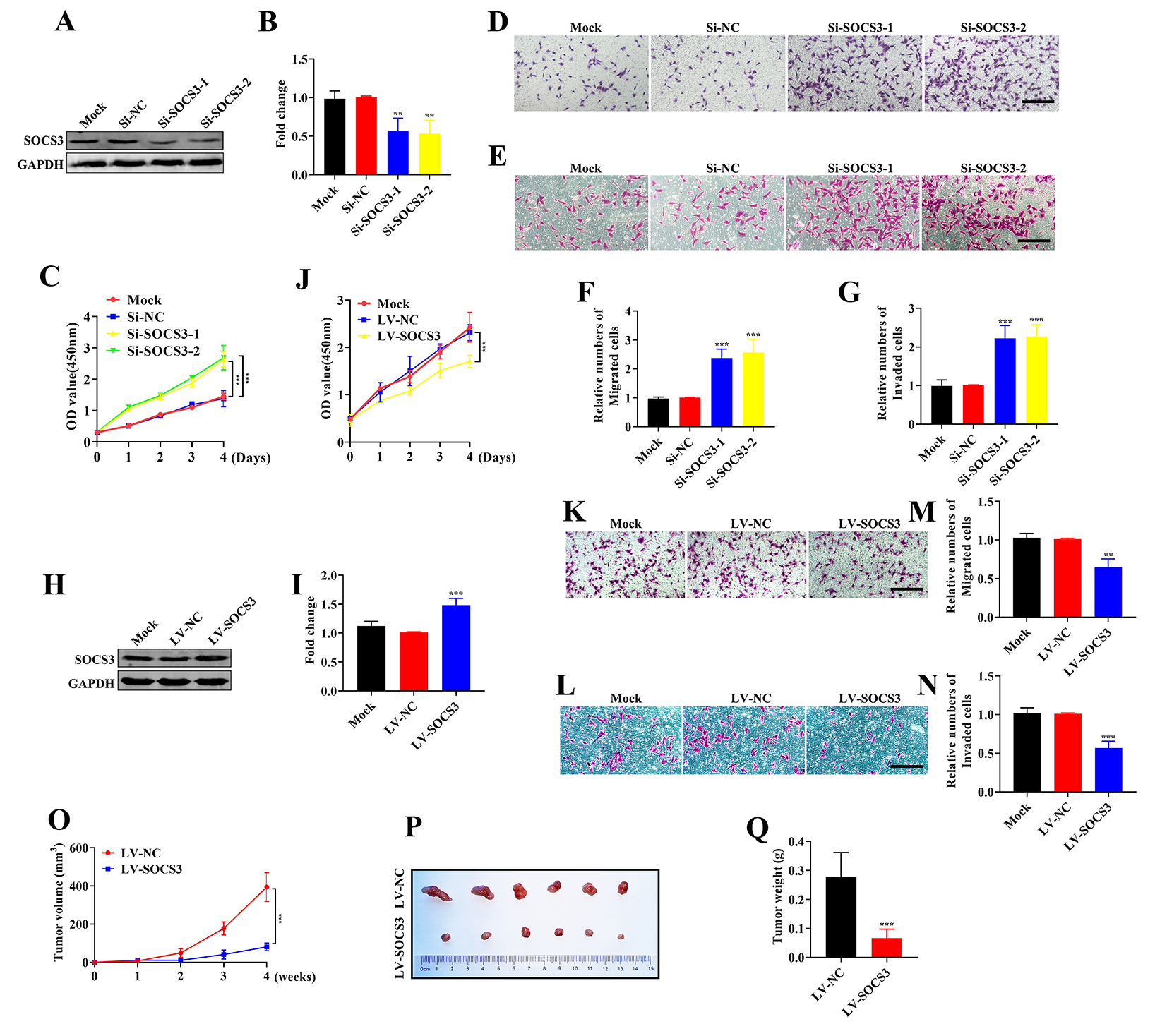


**Fig. S10 SOCS3 inhibits the proliferation, migration, and invasion of UM cell in vitro and in vivo.**

**(A-B)** Western blotting analysis of SOCS3 in UM cell with SOCS3 knockdown. The datas were analyzed using one-way ANOVA with Bonferroni. **(C-G)** Analysis of the proliferation, migration, and invasion of UM cell with SOCS3 knockdown. C was analyzed using two-way ANOVA with Bonferroni. D-G was analyzed using one-way ANOVA with Bonferroni. **(H-I)** Western blotting analysis of SOCS3 in UM cell overexpressing SOCS3. The datas were analyzed using one-way ANOVA with Bonferroni. **(J-N)** Proliferation, migration, and invasion analyses of UM cell overexpressing SOCS3. J was analyzed using two-way ANOVA with Bonferroni. K-N was analyzed using one-way ANOVA with Bonferroni. **(O)** The volume of tumors formed in the LV-NC group and LV-SOCS3 group. The datas were analyzed using two-way ANOVA with Bonferroni. **(P)** Photographic images of tumors from the LV-NC group and LV-SOCS3 group. **(Q)** The weight of tumors formed in the LV-NC group and LV-SOCS3 group. The datas were analyzed using Student’s t-test. (A-Q: C918 cell; scale bar: 100 µm; data are presented as the mean ± SD; n = 3–6; ns: no significant difference, *p < 0.05, **p < 0.01, ***p < 0.001).


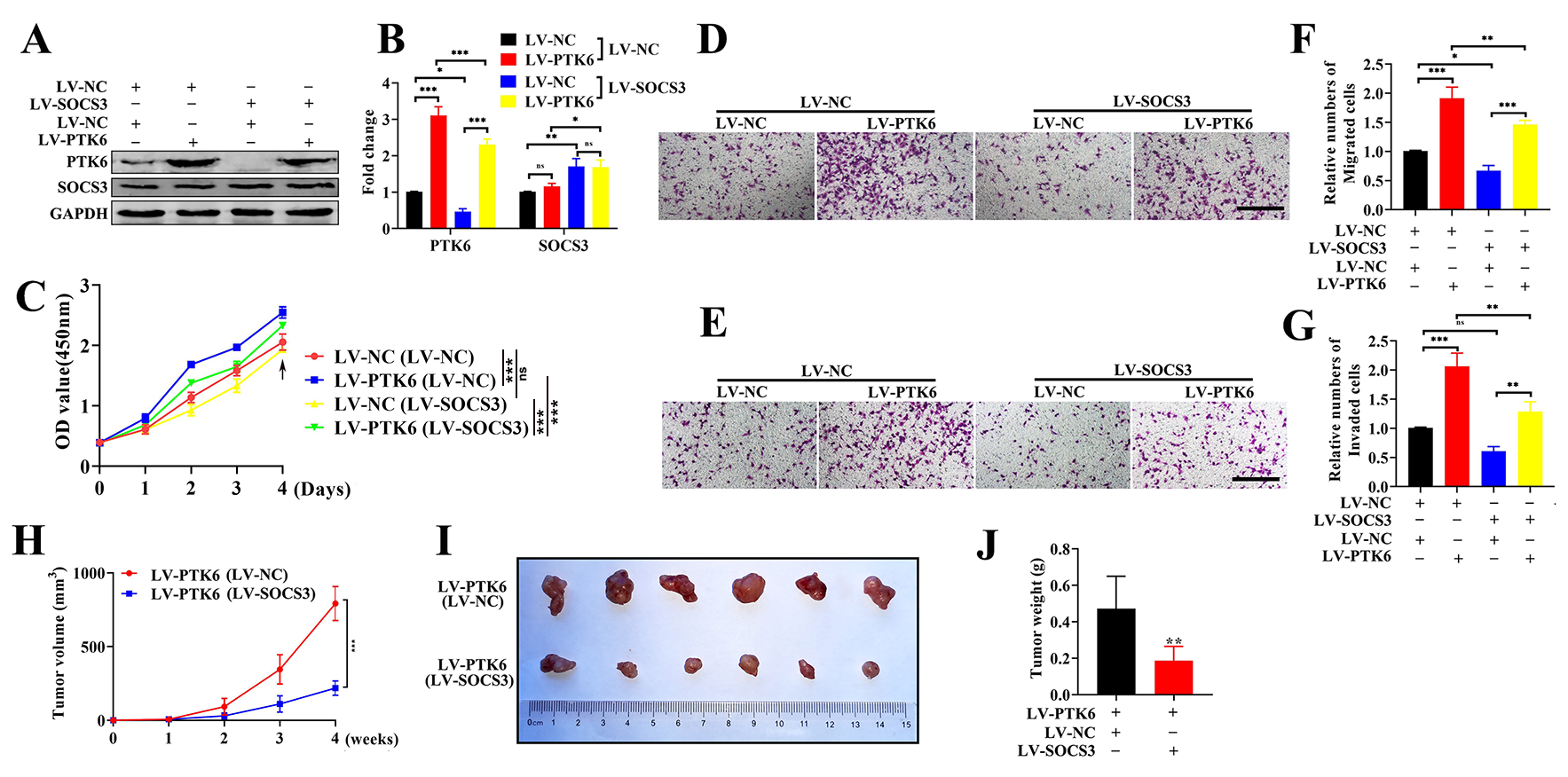


**Fig. S11** **Overexpressing SOCS3 can partially attenuate the PTK6-induced promotion of UM cell proliferation, migration, and invasion in vitro and in vivo. (A-B)** Western blotting analysis of SOCS3 and PTK6 in the LV-NC group or LV-PTK6 group supplemented with LV-NC and LV-SOCS3. (**C-G)** Proliferation, migration, and invasion analysis of the LV-NC group or LV-PTK6 group treated with LV-NC and LV-SOCS3. The above datas were analyzed using two-way ANOVA with Bonferroni. **(H)** The volume of tumors formed in the LV-PTK6/LV-NC group and LV-PTK6/LV-SOCS3 group. The datas were analyzed using two-way ANOVA with Bonferroni. **(I)** Photographs of tumors from the LV-PTK6/LV-NC group and LV-PTK6/LV-SOCS3 group. **(J)** The weight of tumors formed in the LV-PTK6/LV-NC group and LV-PTK6/LV-SOCS3 group. The datas were analyzed using Student’s t-test. (A-J: C918 cell; scale bar: 100 µm; data are presented as the mean ± SD; n = 3–6; ns: no significant difference, *p < 0.05, **p < 0.01, ***p < 0.001).


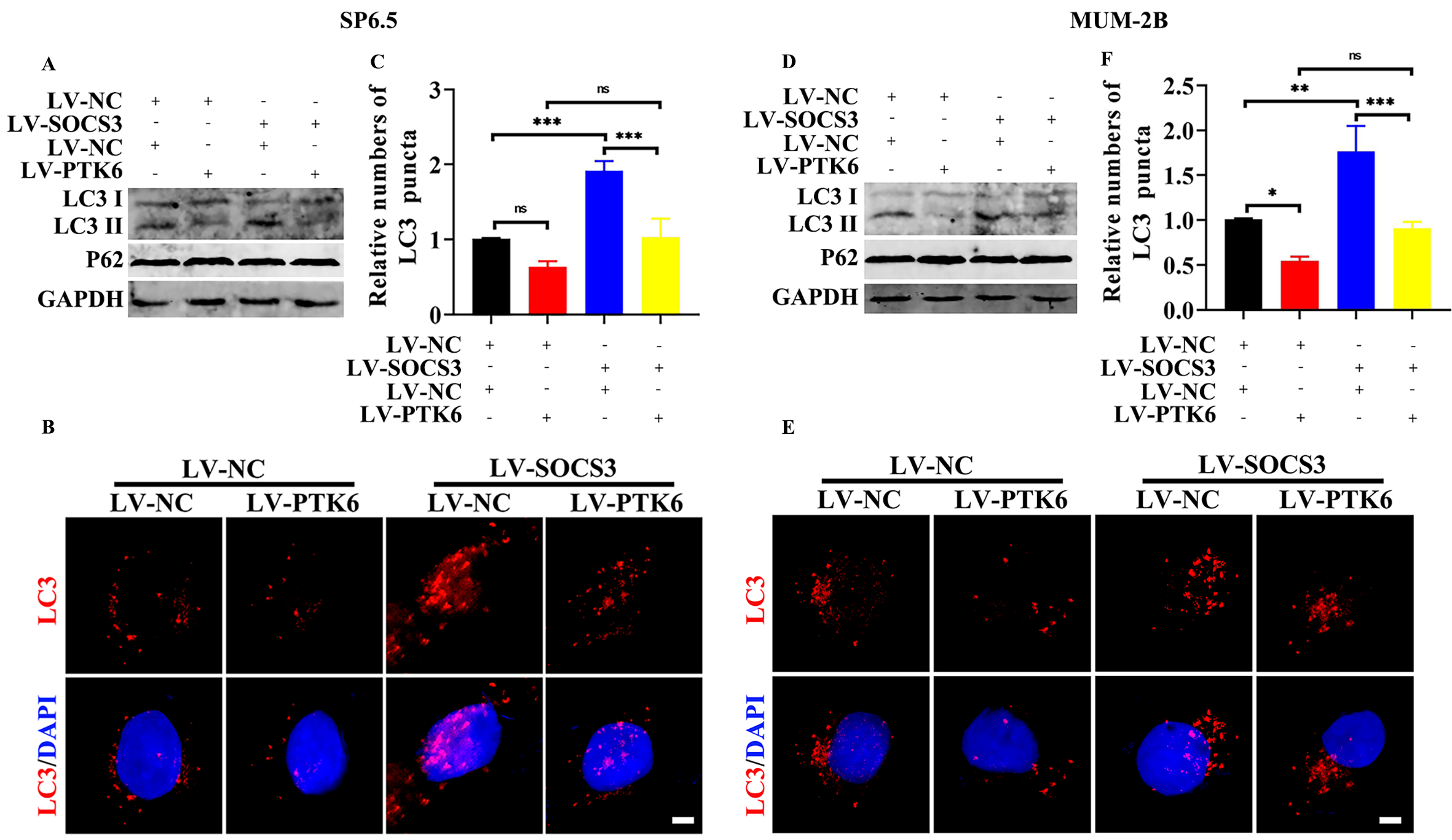


**Fig. S12** **Overexpressing SOCS3 can partially** **rescue the PTK6-inhibited autophagy in UM cells. (****A and D)** Western blotting analysis of LC3 and P62 in the LV-NC group or LV-PTK6 group supplemented with LV-NC and LV-SOCS3. (**B and E)** Immunofluorescence analysis of autophagosomes in UM cell. **(C and F)** The statistical results of (B) and (E). The datas were analyzed using two-way ANOVA with Bonferroni. (A-C: SP6.5 cell; D-F: MUM-2B cell; scale bar: 20 µm; data are presented as the mean ± SD; n = 3; ns: no significant difference, *p < 0.05, **p < 0.01, ***p < 0.001).


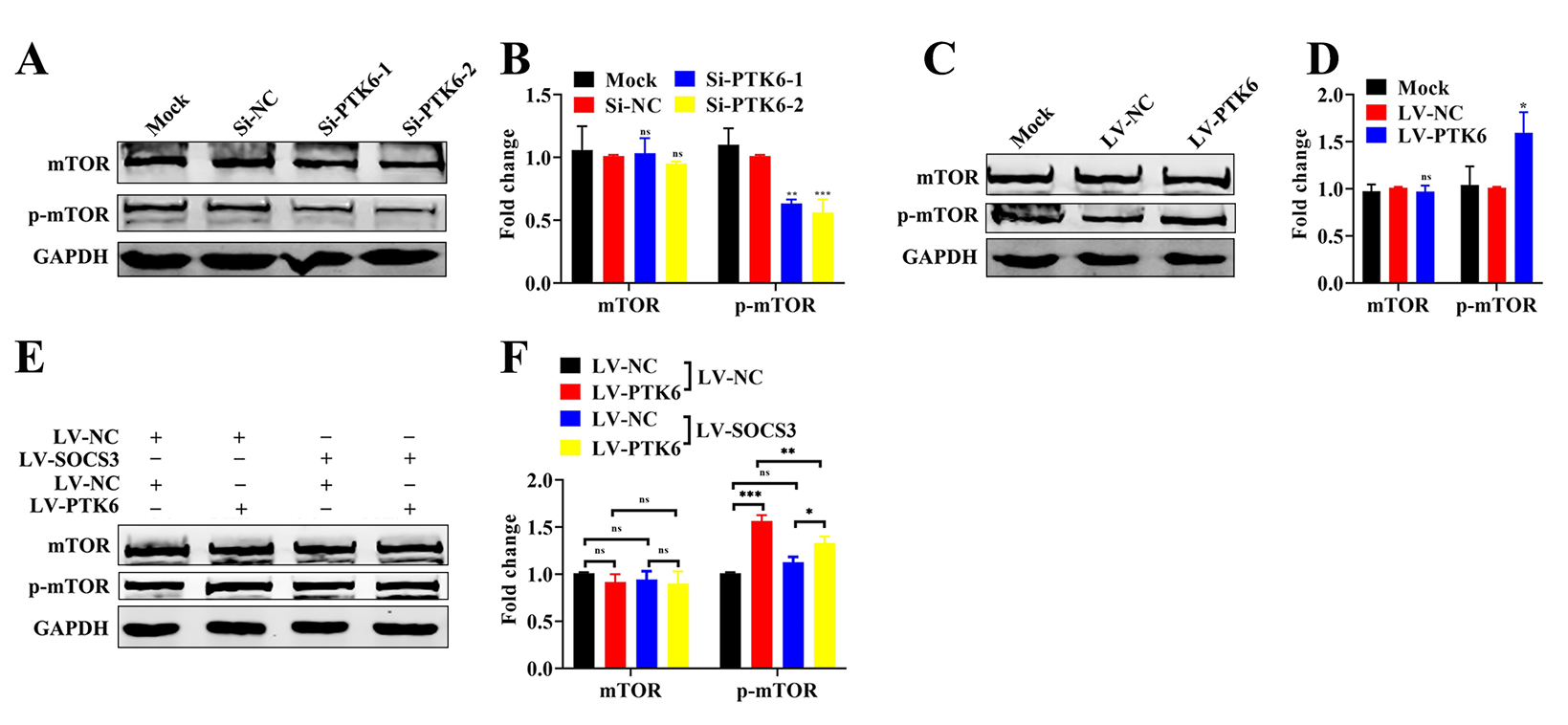


**Fig. S13 PTK6 promotes mTOR phosphorylation.**

**(A-B)** Western blotting analysis of mTOR and p-mTOR in UM cell with PTK6 knockdown. **(C-D)** Western blotting analysis of mTOR and p-mTOR in UM cell overexpressing PTK6. The above datas were analyzed using one-way ANOVA with Bonferroni. **(E-F)** Western blotting analysis of mTOR and p-mTOR in the four cell lines: LV-NC/LV-NC, LV-PTK6/LV-NC, LV-NC/LV-SOCS3, and LV-PTK6/LV-SOCS3. The datas were analyzed using two-way ANOVA with Bonferroni. (A-F: C918 cell; data are presented as the mean ± SD; n = 3; ns: no significant difference, *p < 0.05, **p < 0.01, ***p < 0.001).


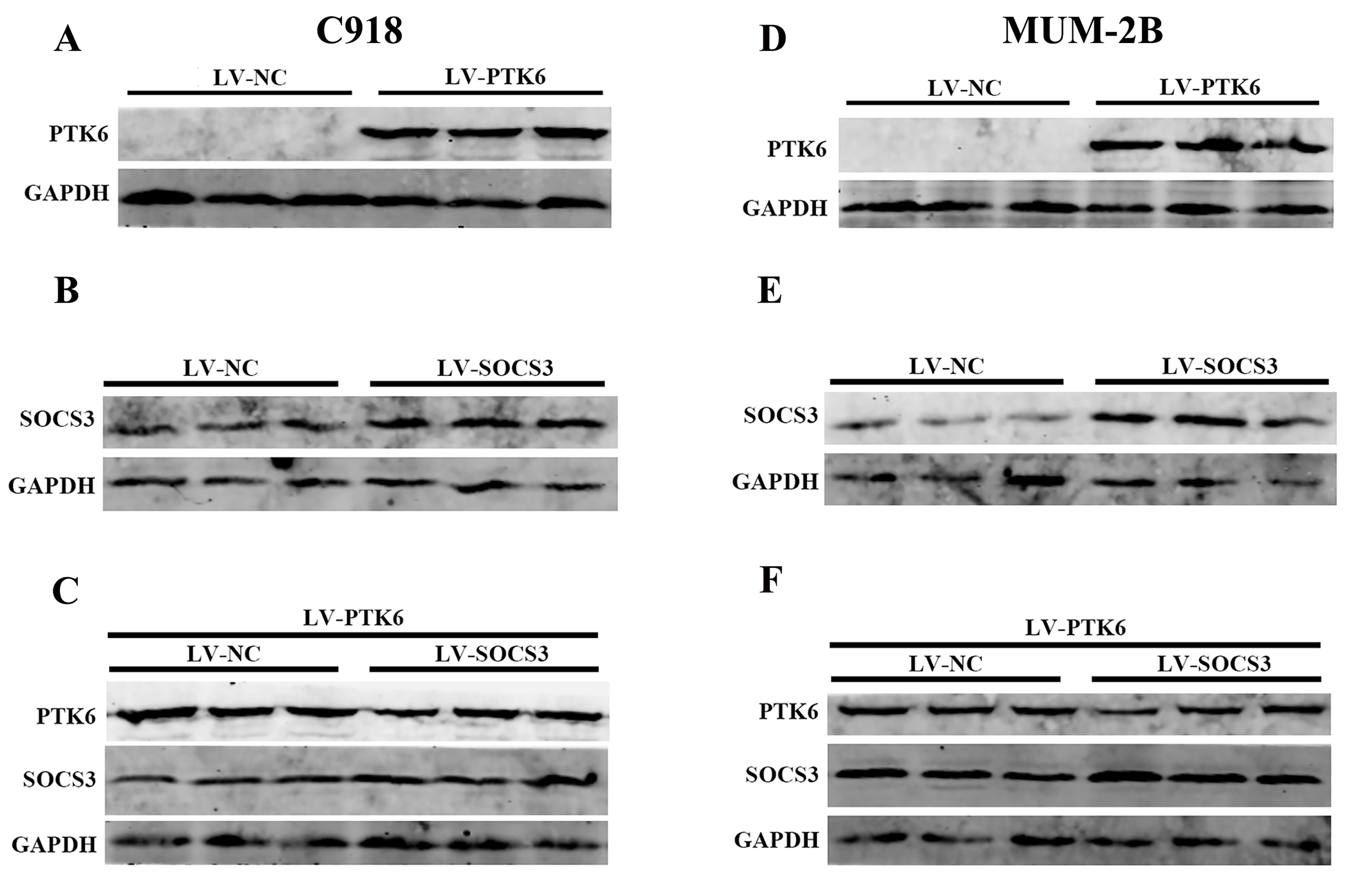


**Fig. S14 The expression of PTK6 and SOCS3 in vivo.**

**(A and D)** Western blotting of PTK6 in tumors formed in the LV-NC group and LV-PTK6 group. **(B and E)** Western blotting of SOCS3 in tumors formed in the LV-NC group and LV-SOCS3 group. **(C and F)** Western blotting of PTK6 and SOCS3 in tumors formed in the LV-PTK6/LV-NC group and LV-PTK6/LV-SOCS3 group. (A-C: C918 cell; D-F: MUM-2B cell).


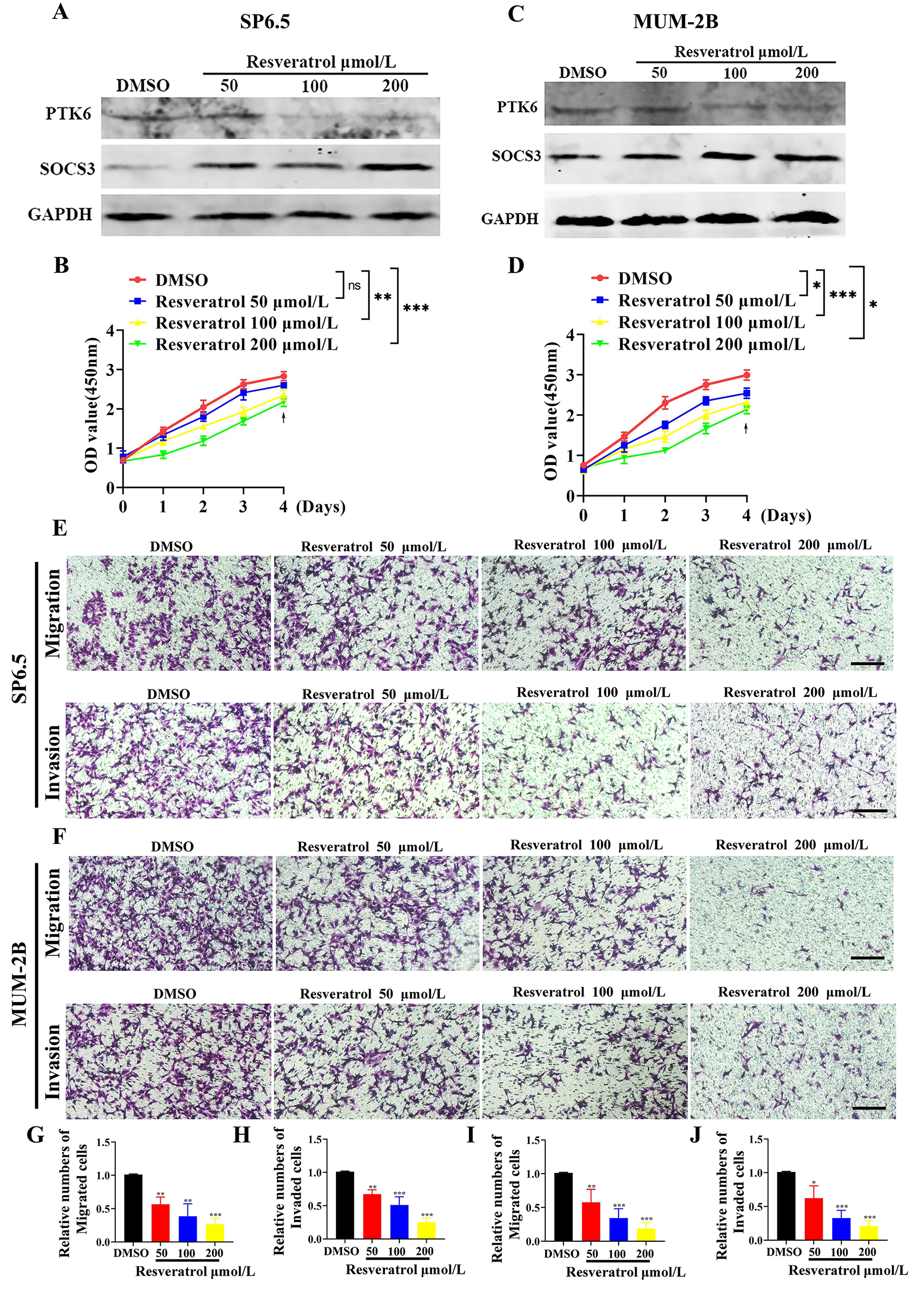


**Fig. S15** **Resveratrol can inhibit the proliferation, migration, and invasion of UM cell by upregulating SOCS3 and inhibiting PTK6 expression.**

**(A and C)** Western blotting analysis of PTK6 and SOCS3 in UM cell with resveratrol treatment. **(B and D-J)** Analysis of the proliferation, migration, and invasion of UM cell with resveratrol treatment. The datas were analyzed using one-way ANOVA with Bonferroni. (A-B, E, G-H: SP6.5 cell; C-D, F, I-J: MUM-2B cell; scale bar: 100 µm; data are presented as the mean ± SD; n = 3; ns: no significant difference, *p < 0.05, **p < 0.01, ***p < 0.001).


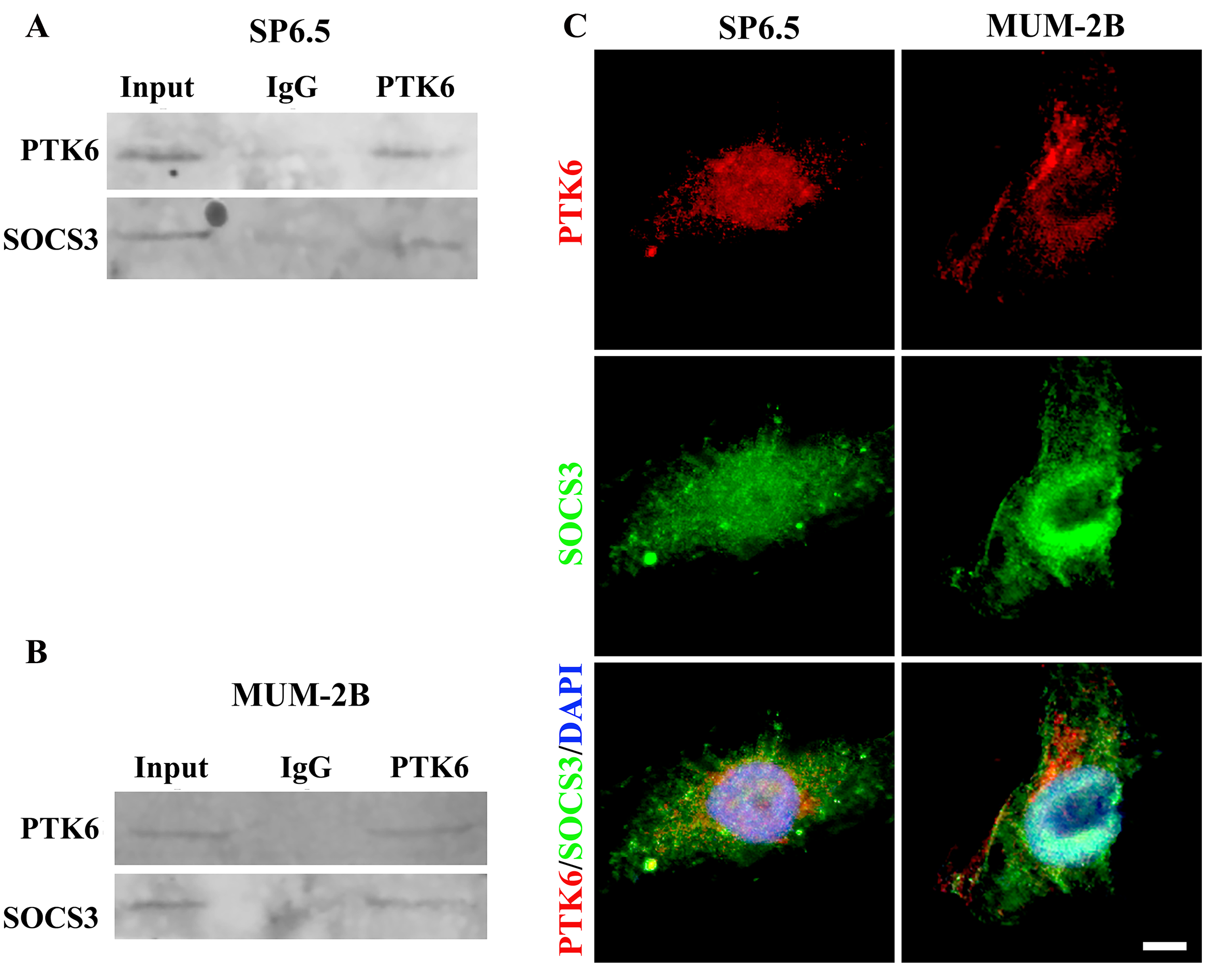


**Fig. S16** **The endogenic PTK6 can bind to SOCS3**

**(A)** Coimmunoprecipitation analysis of PTK6 and SOCS3 in UM cells. **(B)** Co-immunofluorescence analysis of PTK6 and SOCS3 in UM cells. (A: SP6.5 cell; B: MUM-2B cell; scale bar: 20 µm).

**
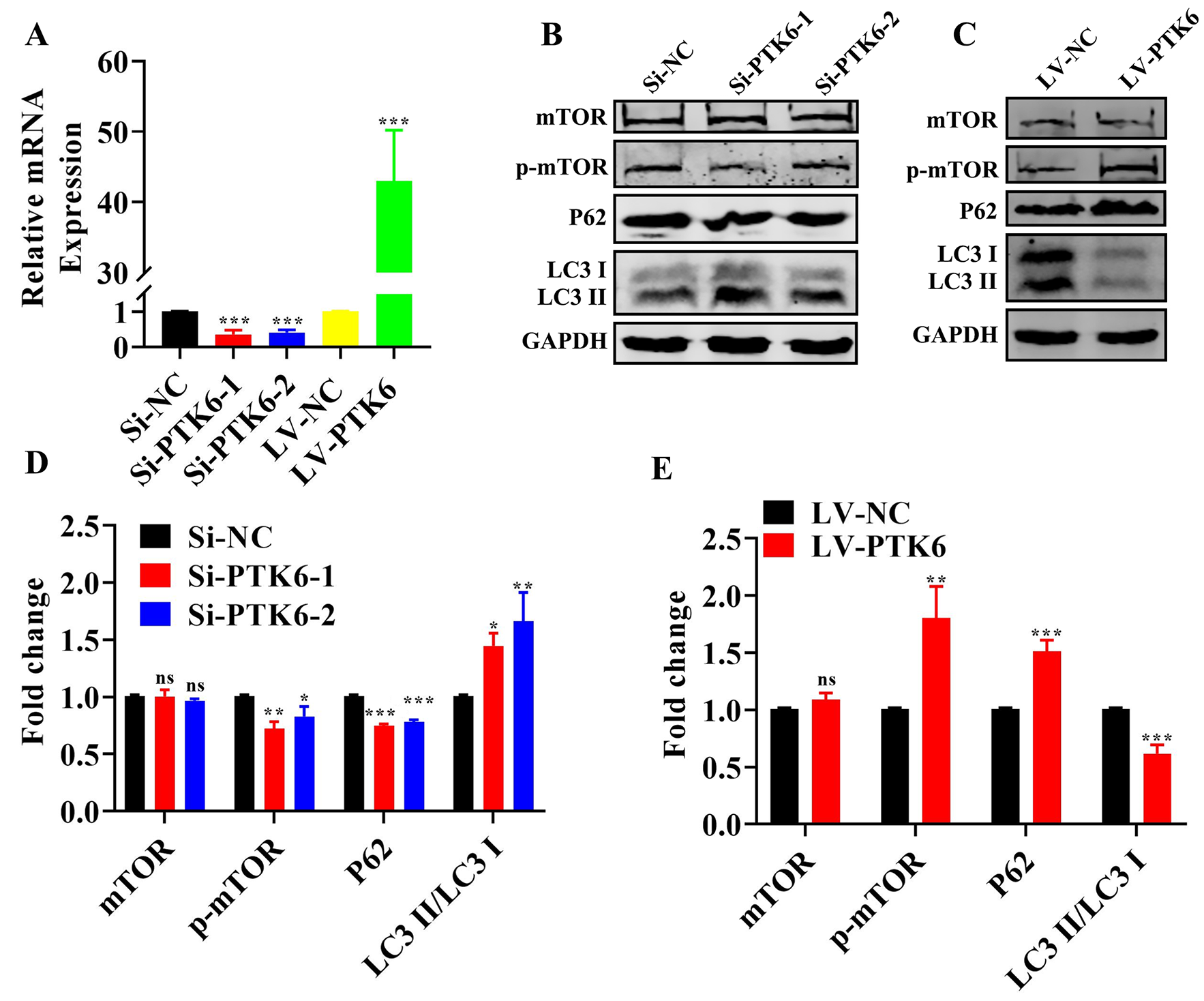
**

**Fig. S17 PTK6 inhibits autophagy by upregulating the phosphorylation of mTOR in ARPE-19 cells.**

**(A)** The expression of *PTK6* mRNA in ARPE-19 cells with PTK6 knockdown or overexpression. **(B and D)** Western blotting analysis of mTOR, p-mTOR, P62, and LC3 in ARPE-19 cells with PTK6 knockdown. **(C and E)** Western blotting analysis of mTOR, p-mTOR, P62, and LC3 in ARPE-19 cells overexpressing PTK6. The above datas were analyzed using one-way ANOVA with Bonferroni. (Data are presented as the mean ± SD; n = 3; ns: no significant difference, *p < 0.05, **p < 0.01, ***p < 0.001).


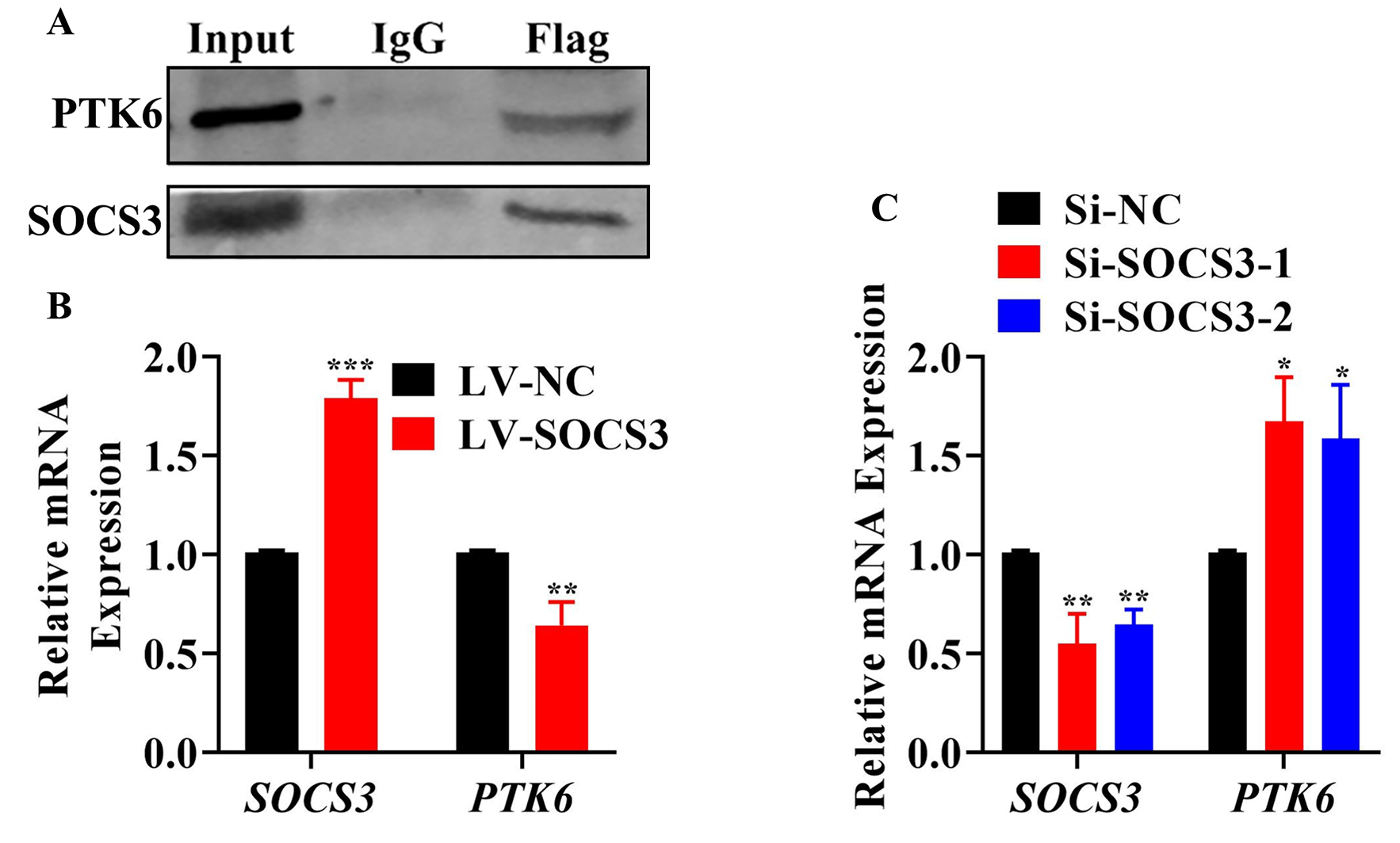


**Fig. S18 SOCS3 binds to PTK6 and inhibits PTK6 expression in ARPE-19 cells.**

**(A)** Coimmunoprecipitation analysis of PTK6 and SOCS3 in ARPE-19 cells overexpressing PTK6. **(B)** The expression of *SOCS3* and *PTK6* mRNA in ARPE-19 cells overexpressing SOCS3. **(C)**The expression of *SOCS3* and *PTK6* mRNA in ARPE-19 cells with SOCS3 knockdown. The above datas were analyzed using one-way ANOVA with Bonferroni. (Data are presented as the mean ± SD; n = 3; *p < 0.05, **p < 0.01, ***p < 0.001).
