# Supplementary figures and images for: PTK6 inhibits autophagy to promote uveal melanoma tumorigenesis by binding to SOCS3 and regulating mTOR phosphorylation
Source: Cell Death Dis. 2023 Jan 23;14(1):55. doi: 10.1038/s41419-023-05590-w (PMC9870980; doi:10.1038/s41419-023-05590-w)

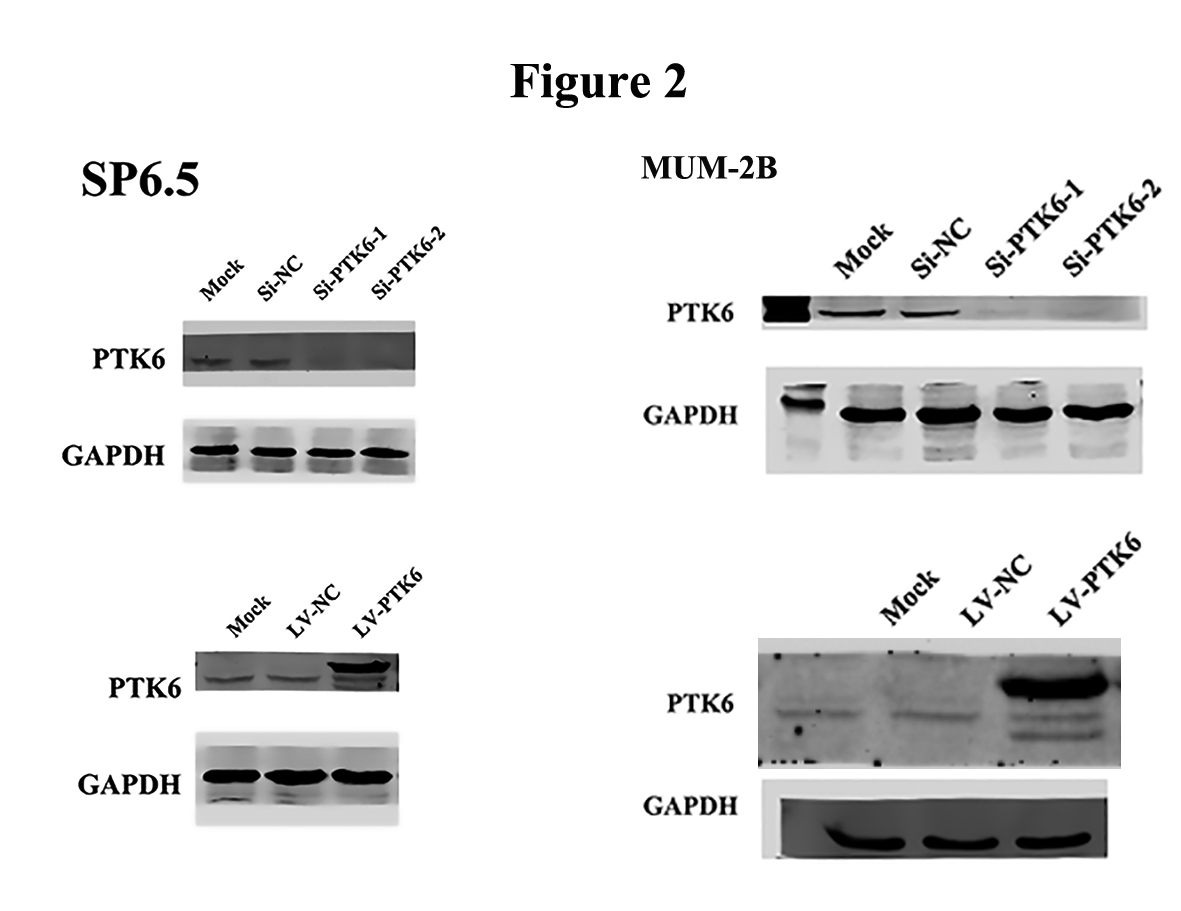

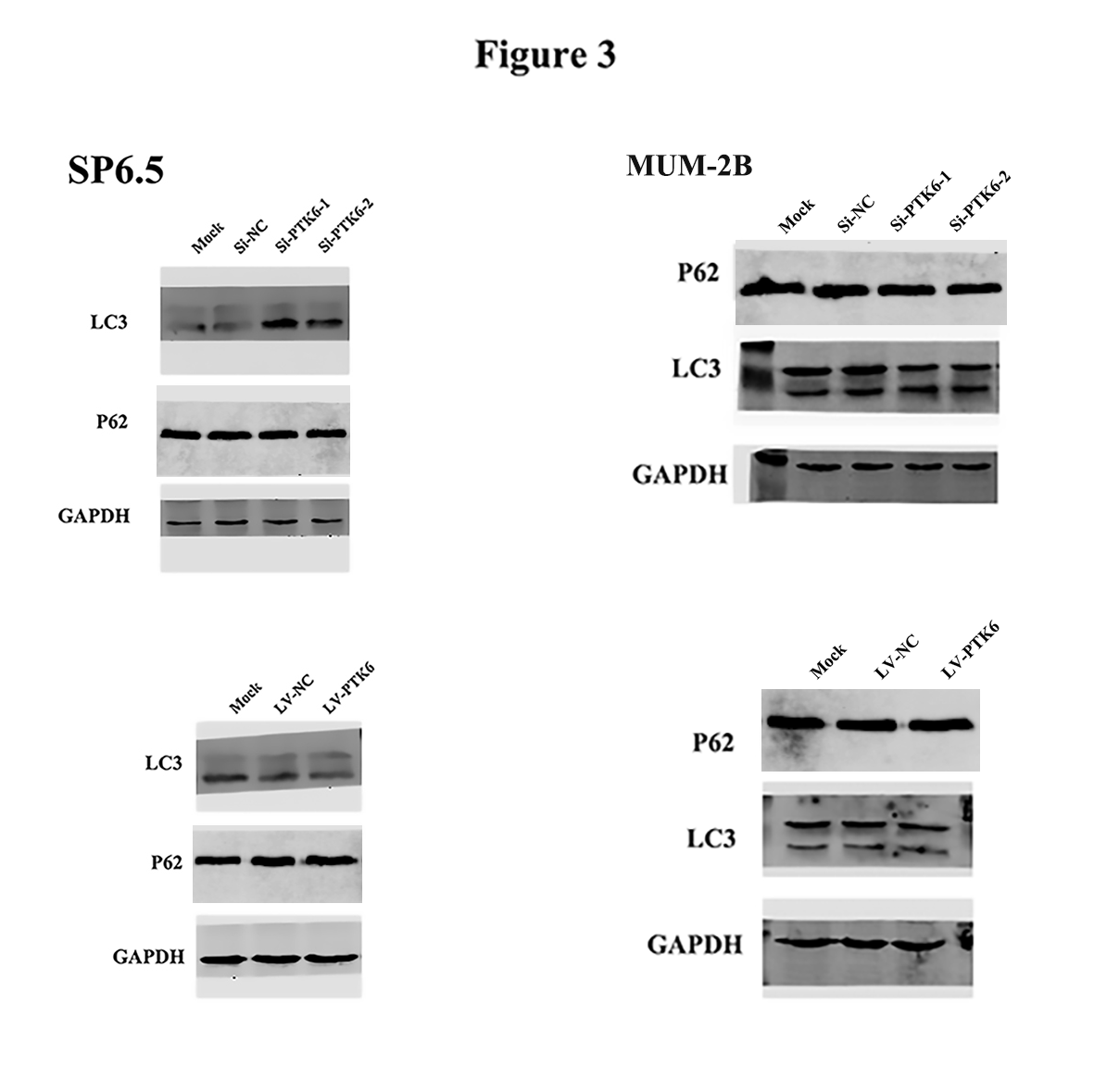

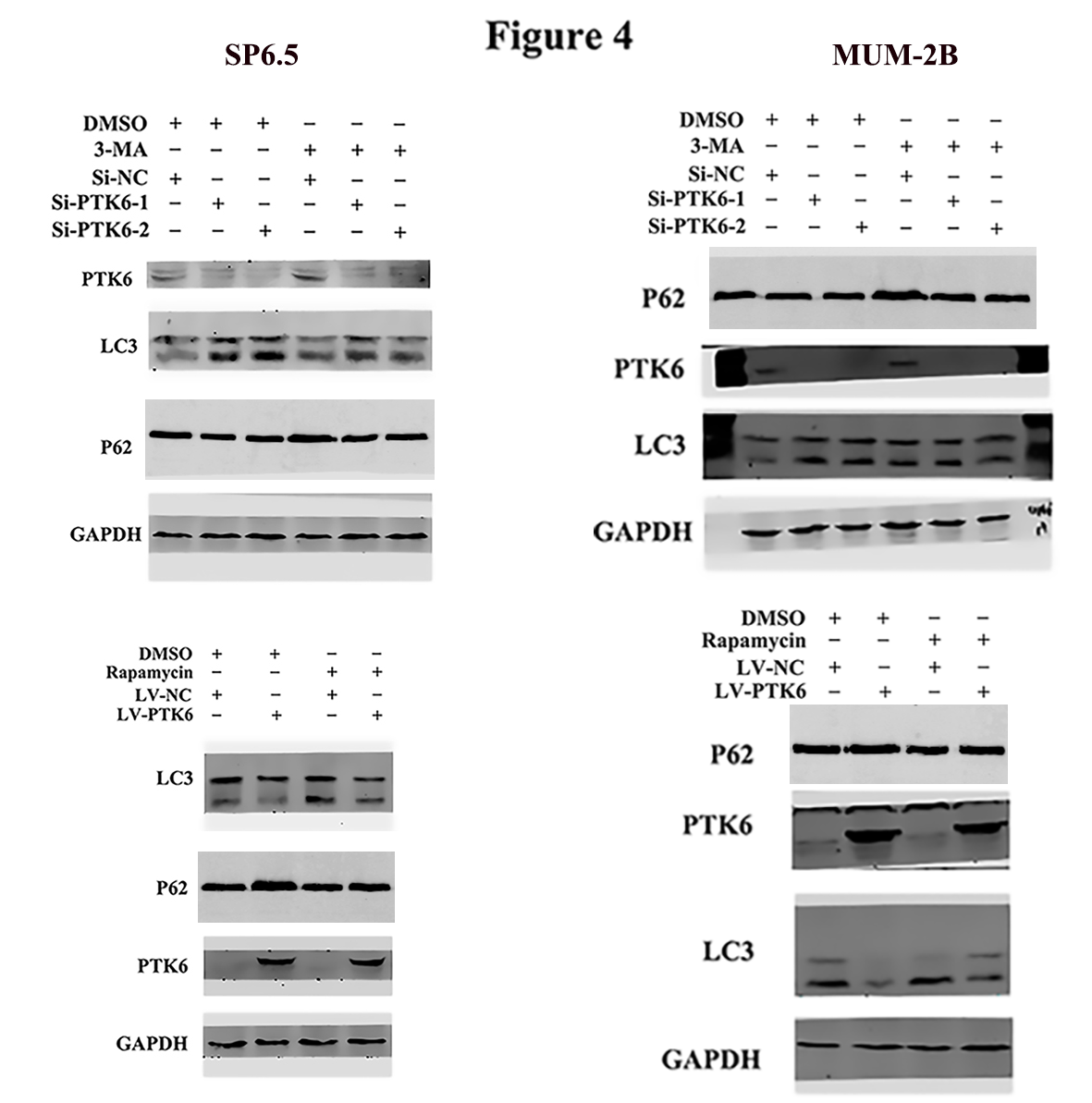

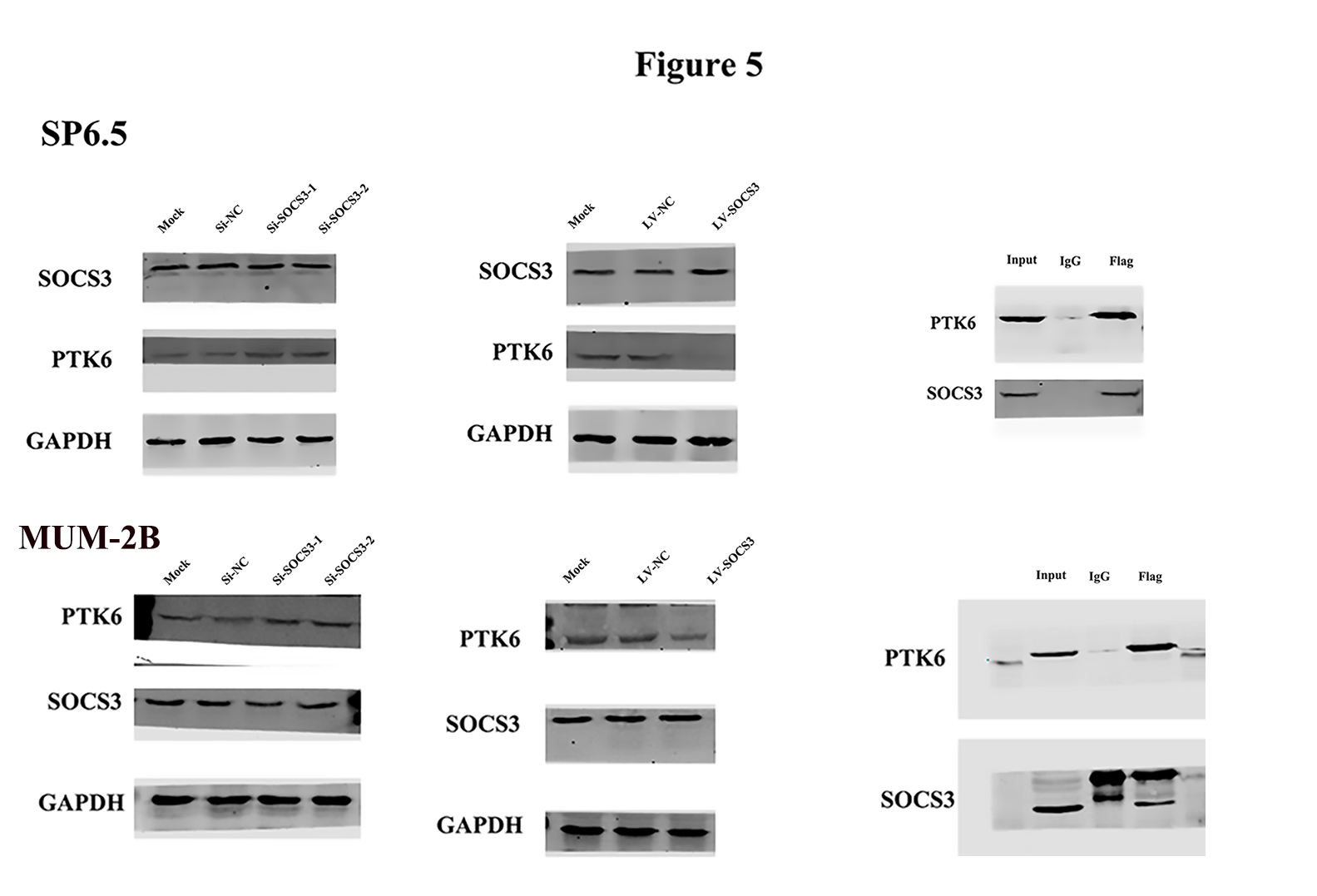

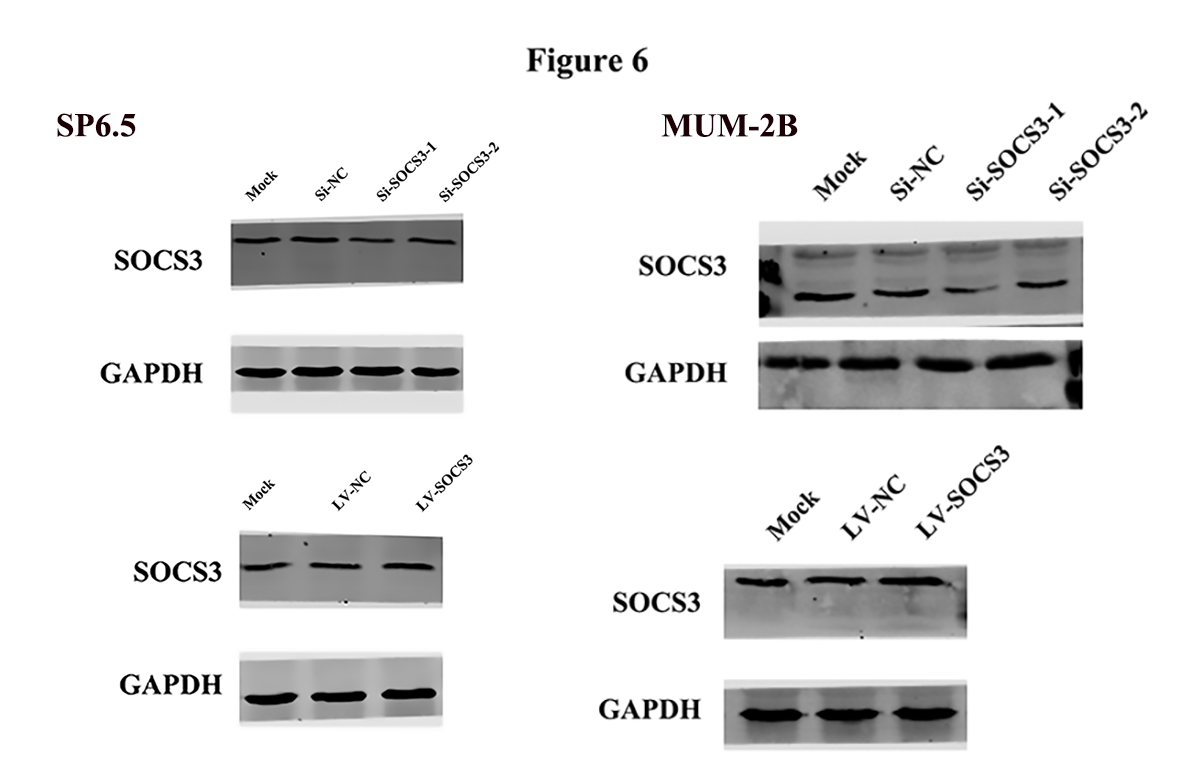


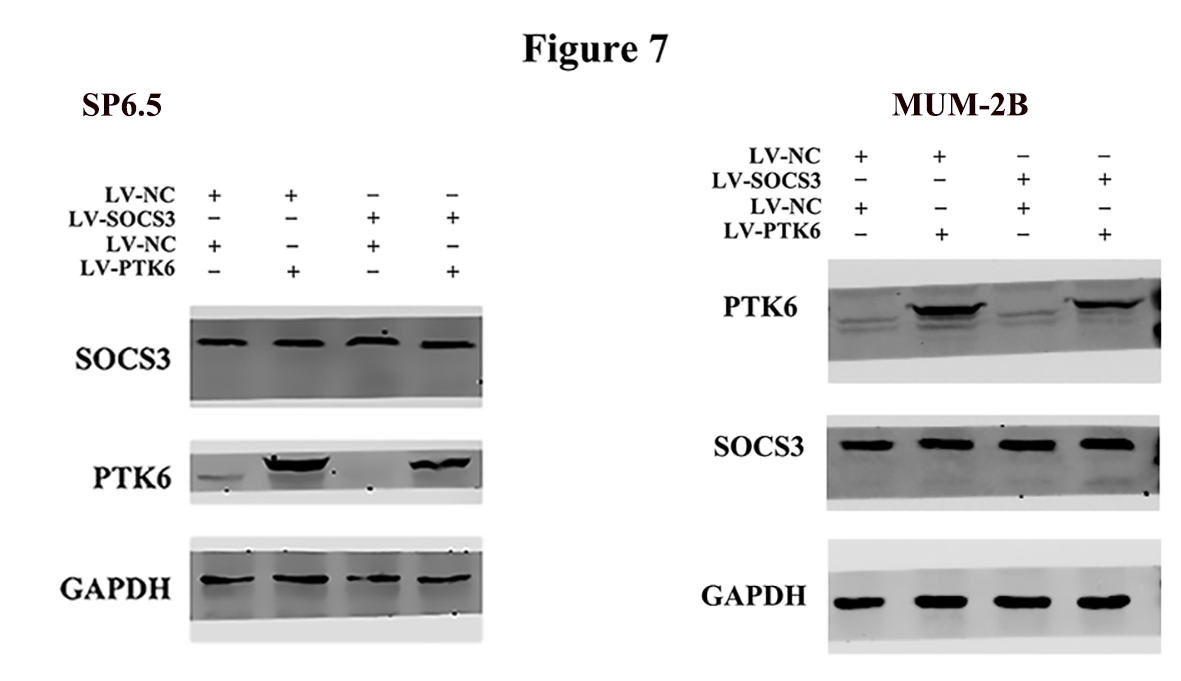

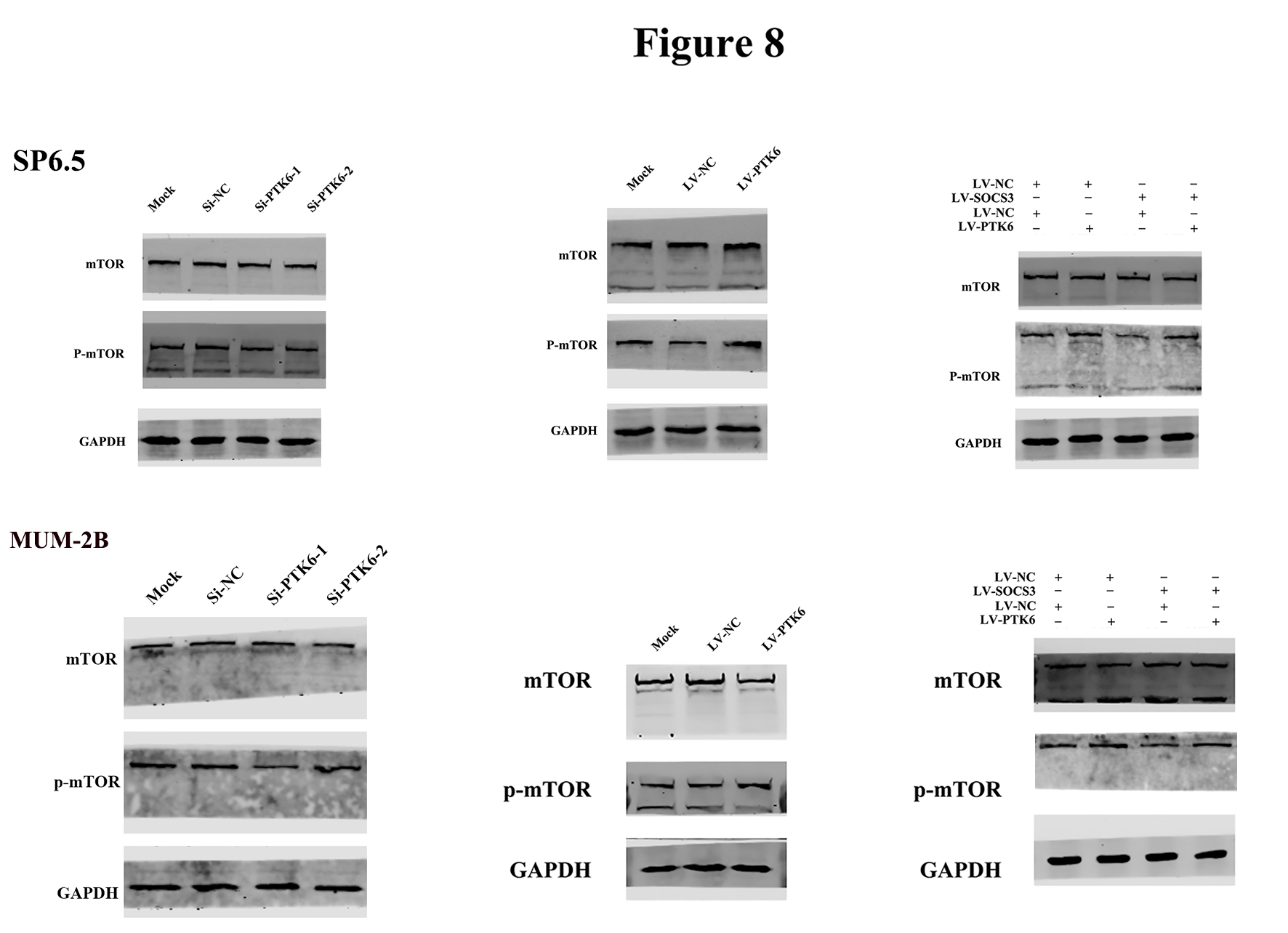

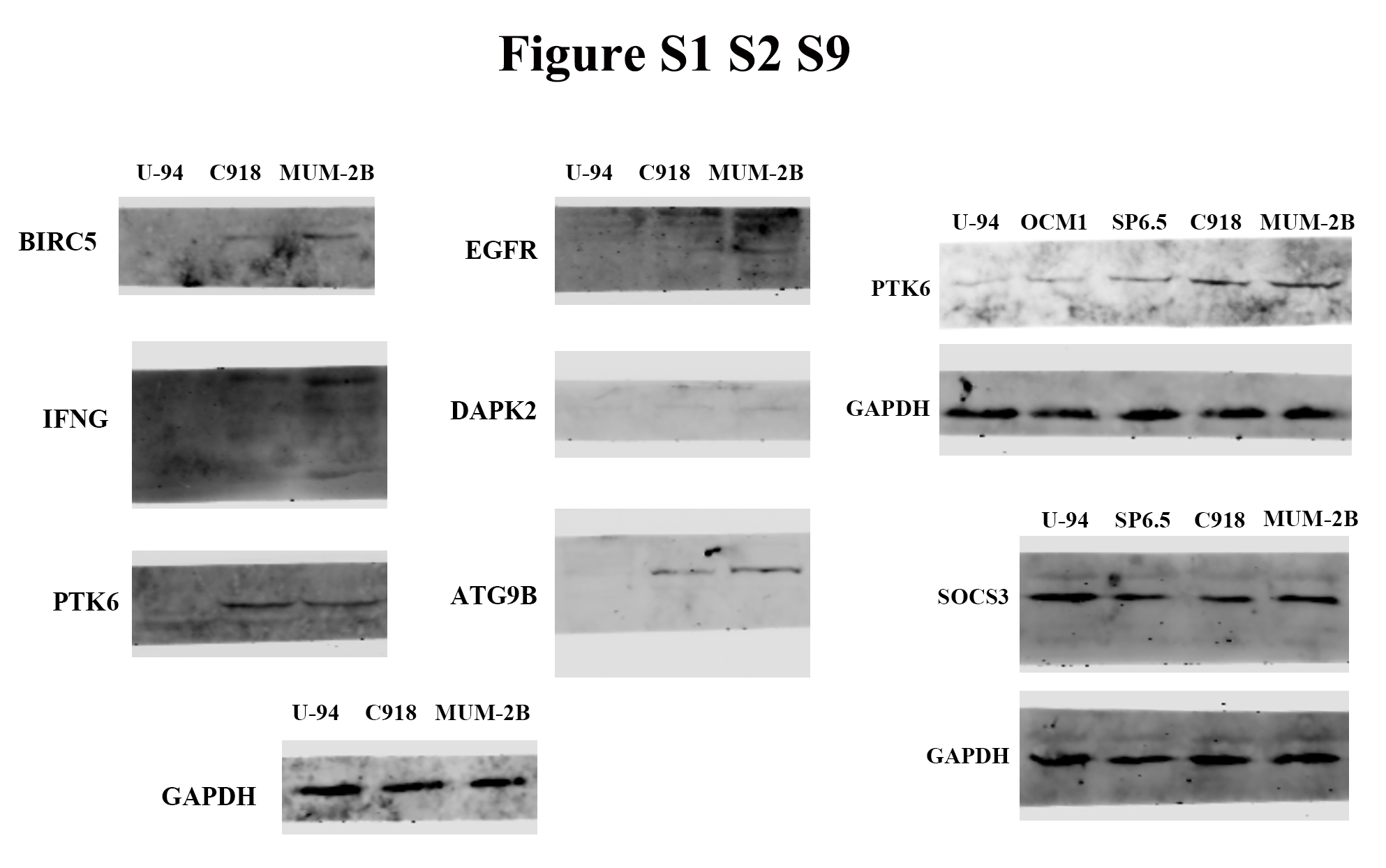

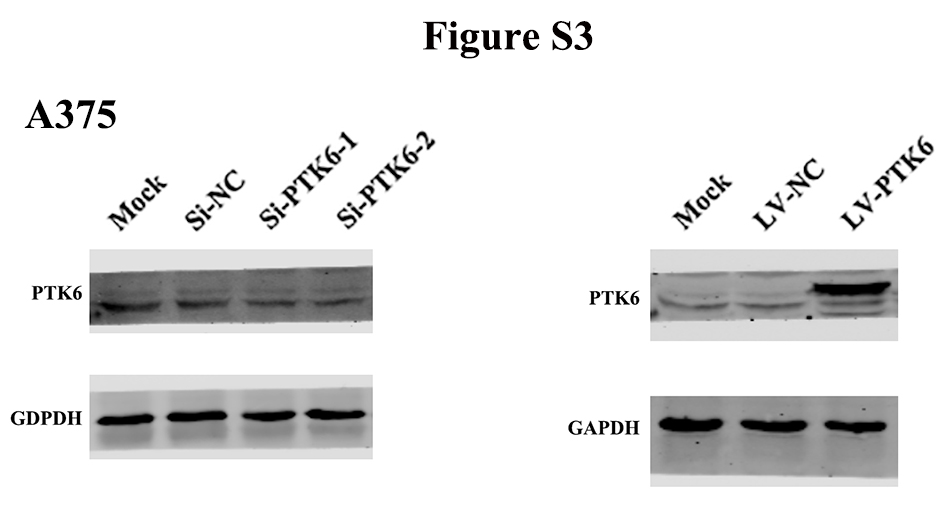

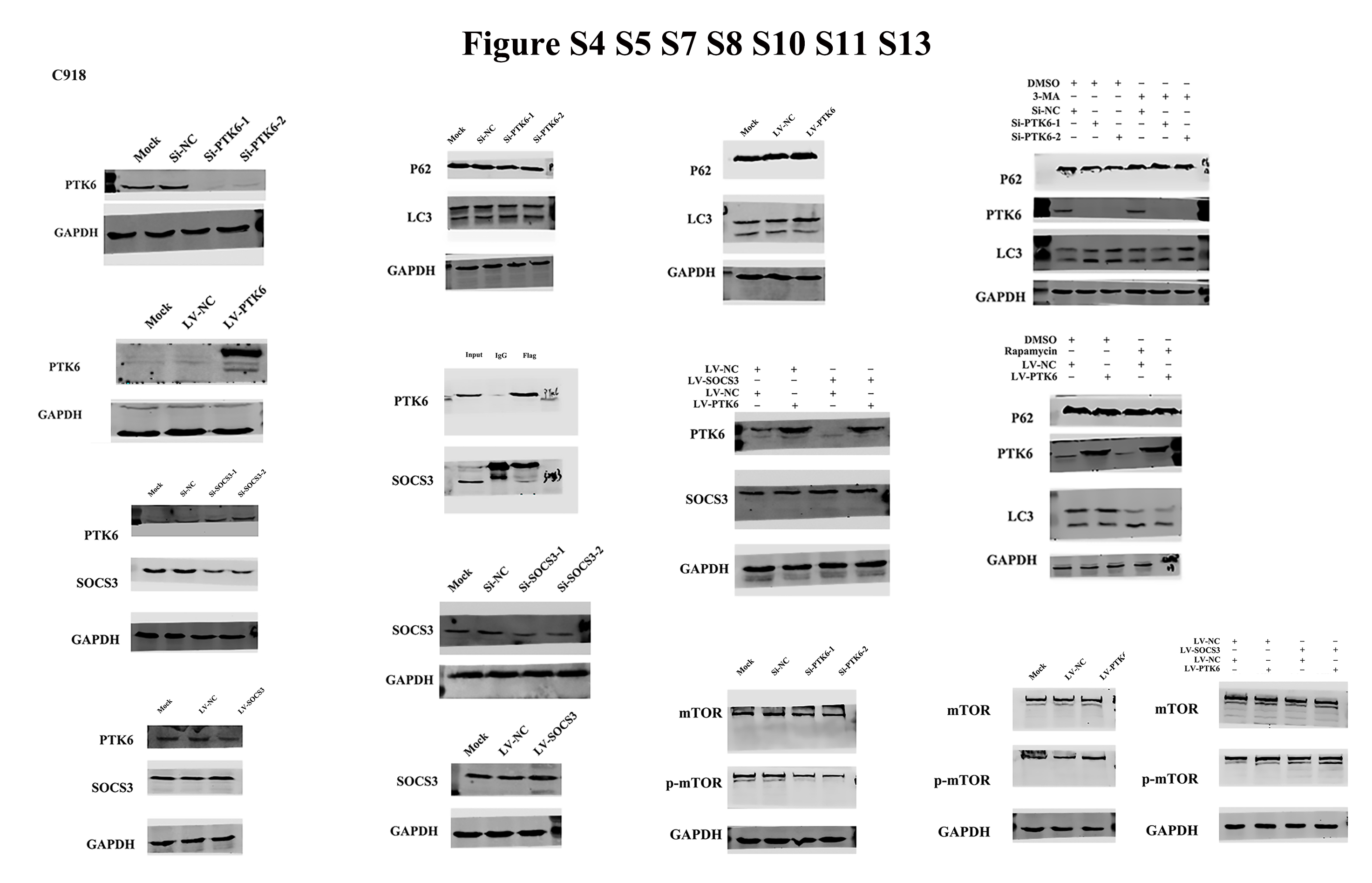

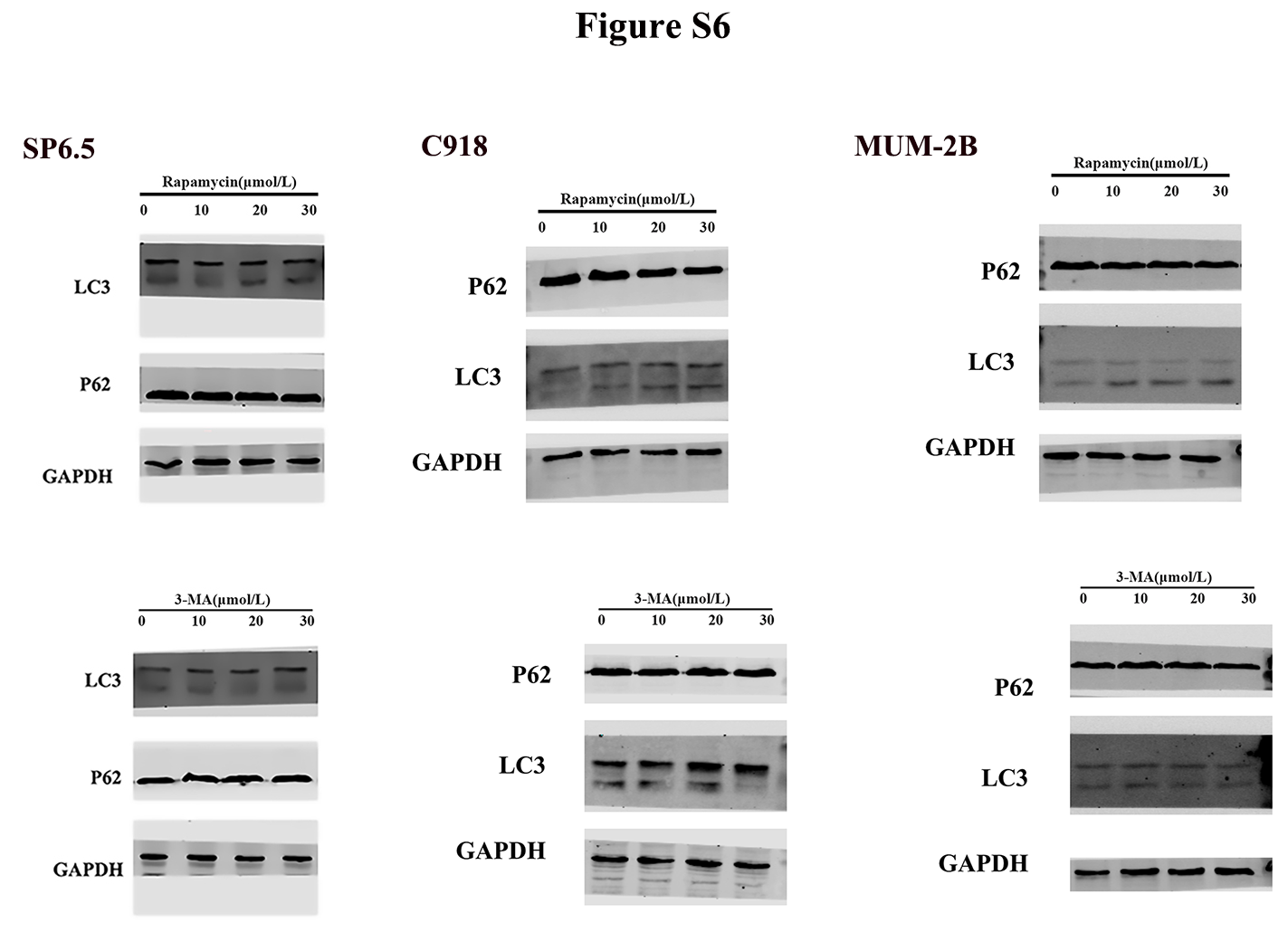

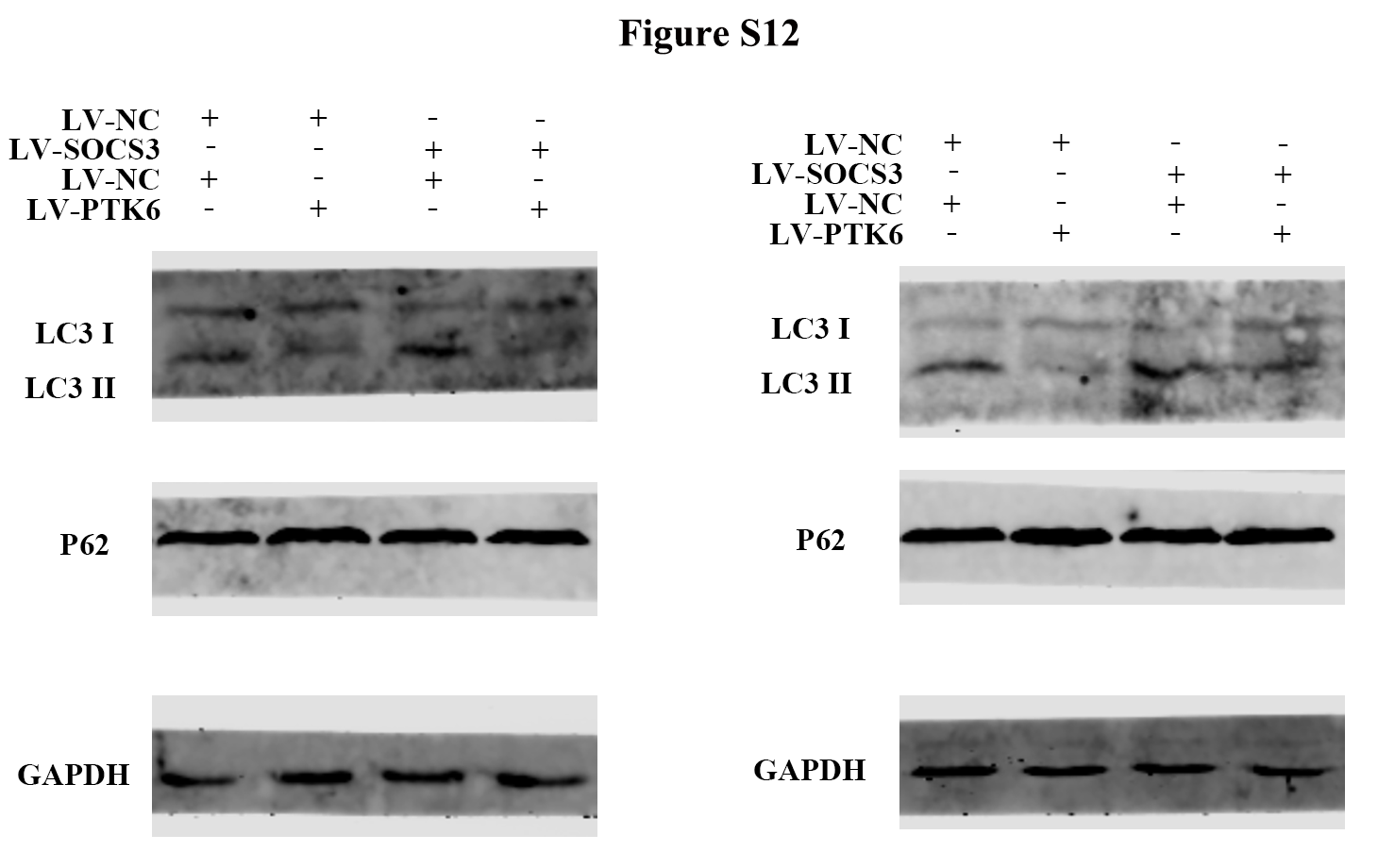

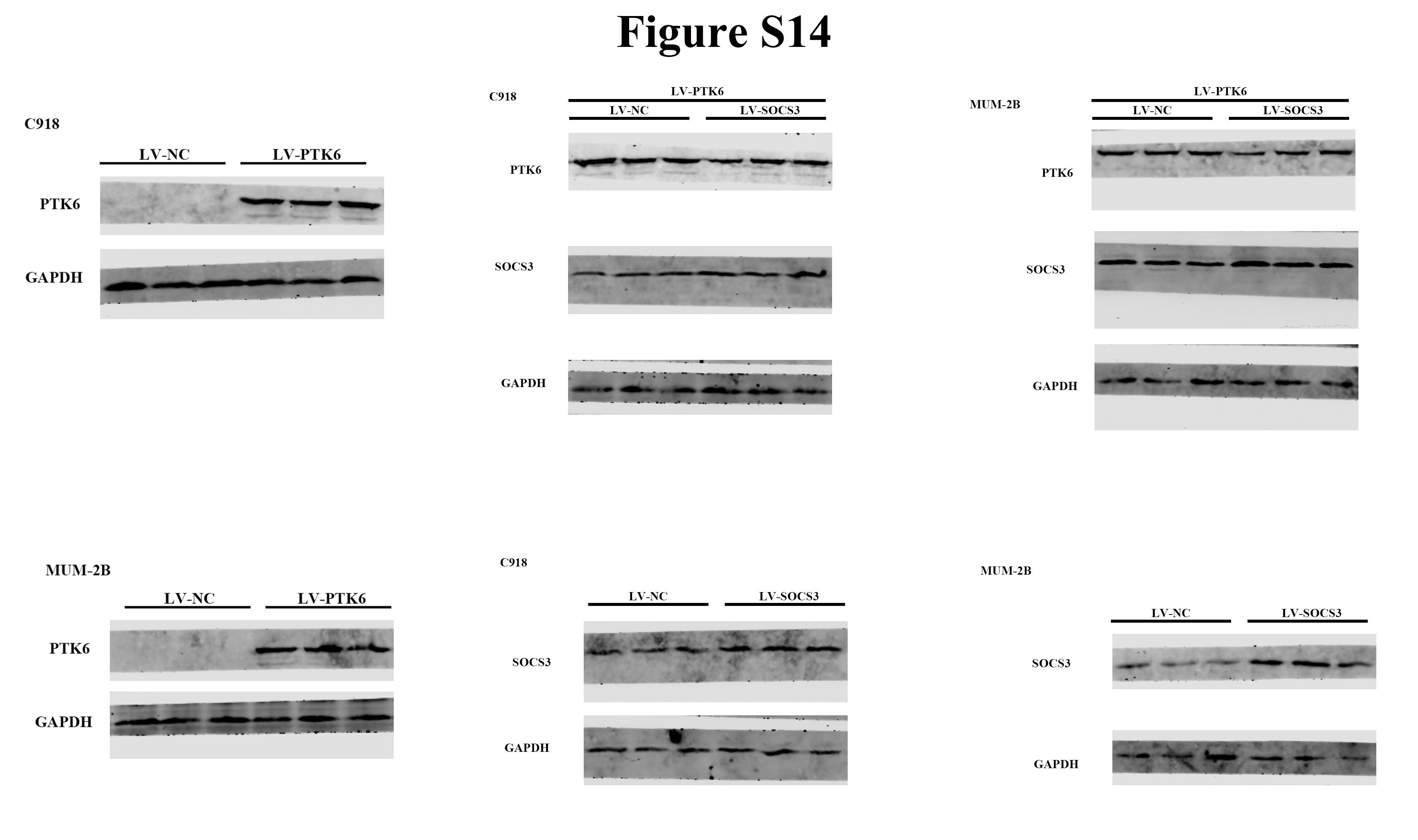

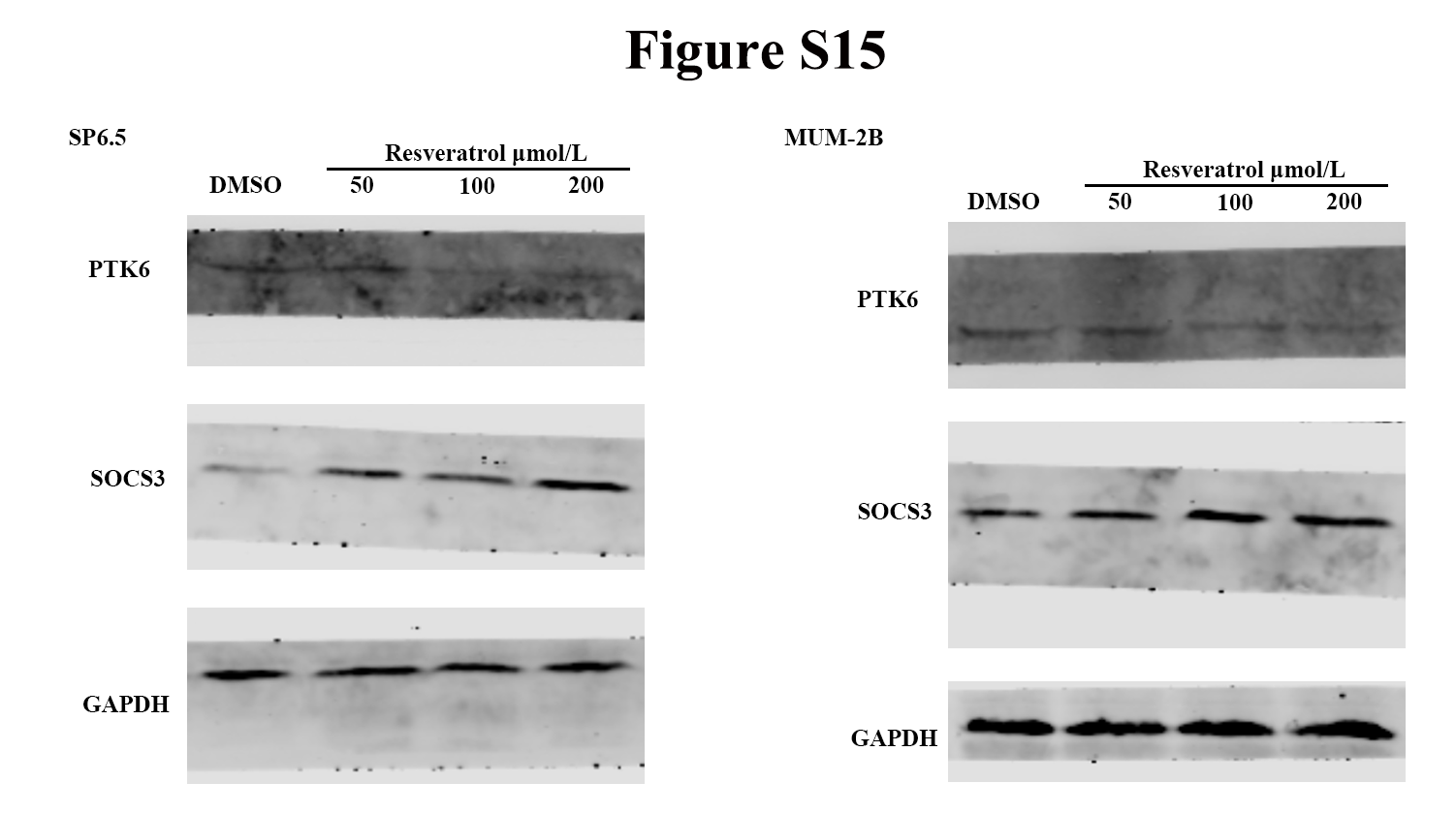

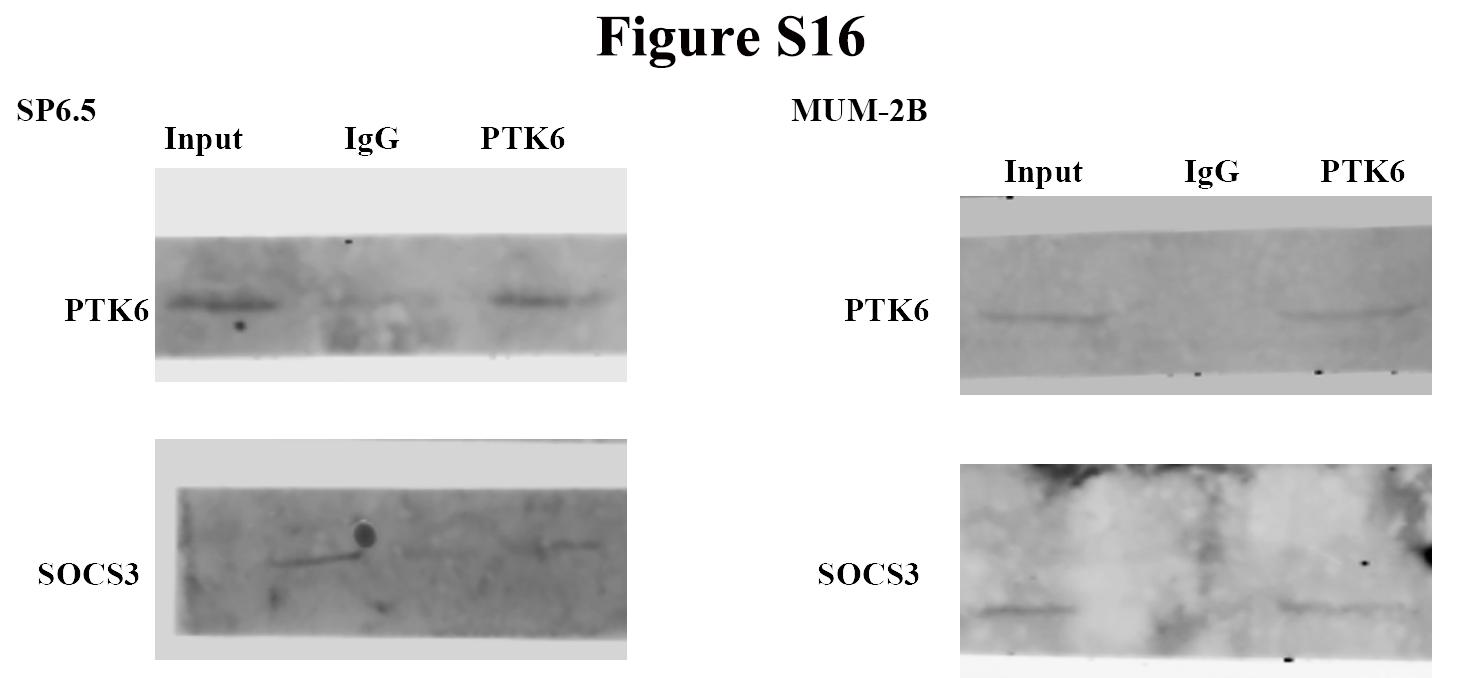

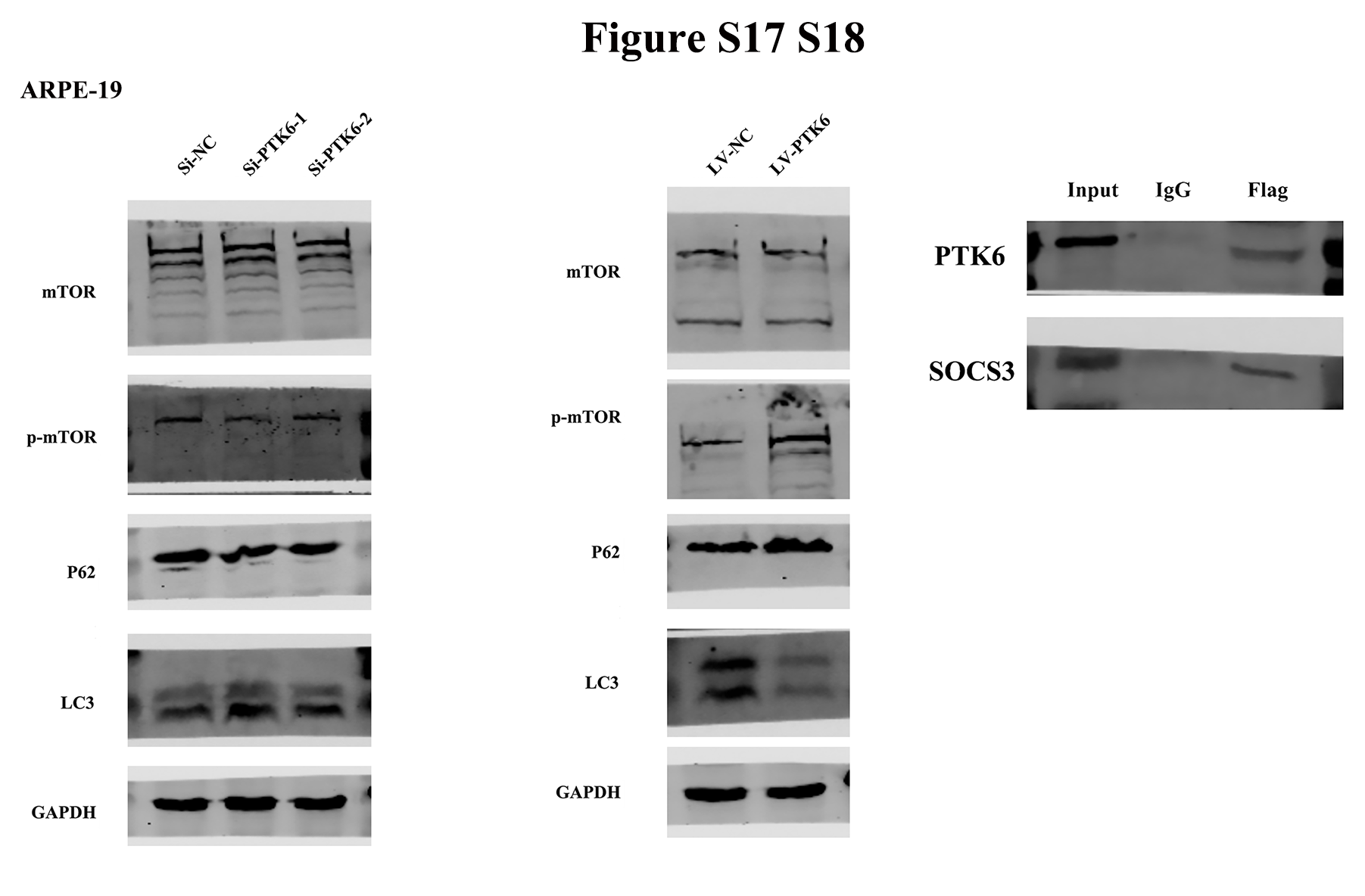

Supplement: Supplementary file 2 — Original Data File [file 41419_2023_5590_MOESM2_ESM.docx]
